# Serum metabolite profiling yields insights into health promoting effect of A. muciniphila in human volunteers with a metabolic syndrome

**DOI:** 10.1080/19490976.2021.1994270

**Published:** 2021-11-23

**Authors:** Clara Depommier, Amandine Everard, Céline Druart, Dominique Maiter, Jean-Paul Thissen, Audrey Loumaye, Michel P. Hermans, Nathalie M. Delzenne, Willem M. de Vos, Patrice D. Cani

**Affiliations:** a Metabolism and Nutrition Research Group, Louvain Drug Research Institute, Walloon Excellence in Life Sciences and BIOtechnology (Welbio), UCLouvain, Université Catholique De Louvain, Brussels, Belgium; b Pôle Edin, Institut De Recherches Expérimentales Et Cliniques, UCLouvain, Université Catholique De Louvain, Brussels, Belgium; c Division of Endocrinology and Nutrition, Cliniques Universitaires St-Luc, Brussels, Belgium; d Laboratory of Microbiology, Wageningen University, Wageningen, The Netherland; e Human Microbiome Research Program, Faculty of Medicine, University of Helsinki, Helsinki, Finland

**Keywords:** *A. muciniphila*, human, obesity, metabolic syndrome, prediabetes, metabolomic, amino-acids, ketone bodies, acylcarnitines

## Abstract

Reduction of *A. muciniphila* relative abundance in the gut microbiota is a widely accepted signature associated with obesity-related metabolic disorders. Using untargeted metabolomics profiling of fasting plasma, our study aimed at identifying metabolic signatures associated with beneficial properties of alive and pasteurized *A. muciniphila* when administrated to a cohort of insulin-resistant individuals with metabolic syndrome. Our data highlighted either shared or specific alterations in the metabolome according to the form of *A. muciniphila* administered with respect to a control group. Common responses encompassed modulation of amino acid metabolism, characterized by reduced levels of arginine and alanine, alongside several intermediates of tyrosine, phenylalanine, tryptophan, and glutathione metabolism. The global increase in levels of acylcarnitines together with specific modulation of acetoacetate also suggested induction of ketogenesis through enhanced β-oxidation. Moreover, our data pinpointed some metabolites of interest considering their emergence as substantial compounds pertaining to health and diseases in the more recent literature.

## Introduction

The past two decades of research in the field of obesity-associated comorbidities were marked by the recognition of the gut microbiota as a key factor in disease etiology.^1^ Efforts to characterize the human gut microbiota in health and disease, encompassing extensive translational research in rodent models, have led to the identification of the Gram-negative bacterium *Akkermansia muciniphila* (*A. muciniphila*) as a next-generation beneficial microbe.^2^ We demonstrated that the bacterium exerts anti-obesogenic and health-promoting effects in mice,^3^ which was subsequently widely confirmed by numerous publications and extended to other metabolic disorders (for the latest reviews see^4–7^). Following the unexpected discovery of pasteurization as a way to amplify its benefits in a similar murine model,^8^ we implemented the first human pilot intervention, called Microbes4U©, consisting of daily ingestion of either alive or pasteurized *A. muciniphila* for 12 weeks. The initial primary objectives of the Microbes4U© study were to evaluate the tolerance, safety, and feasibility of *A. muciniphila* administration in a population with excess body weight suffering from prediabetes and metabolic syndrome. Not only we confirmed these aims but our data also showed several proofs of efficacy of the bacterium with stronger effects of the pasteurized form.^9^ Beneficial impacts were shown on the levels of blood lipids, glycemic parameters such as insulin resistance, hepatic status with reduced blood levels of liver enzymes, and endotoxemia. We also observed some interesting trends regarding anthropometric measurements associated with obesity. Further in-depth toxicological analysis in rats confirmed the safety of the administration of the pasteurized *A. muciniphila*.^10^ Although many metabolic outcomes and potential causal factors were identified in preclinical studies, some pieces of machinery remain to be uncovered. The collection of biological samples during the study allowed us to further explore the mechanisms underlying host–*A. muciniphila* crosstalk in the context of the Microbes4U© study. We notably highlighted that beneficial effects of *A. muciniphila* were independent of any major shift of the circulating endocannabinoidome. However, we identified a potential positive influence on the plasma levels of peroxisome proliferator-activated receptor alpha (PPARα) agonist 2-palmitoyl-glycerol.^1^

As *A. muciniphila* shows such a large effect on a variety of parameters, it is likely to influence a large series of other bioactive molecules. Hence, we were interested in carrying out a metabolomic study on blood samples to identify potential pathways affected by the bacterium as well as novel metabolite signatures associated with the metabolic effects of *A. muciniphila*. In this placebo-controlled interventional study conducted in treatment-naive subjects with prediabetes or metabolic syndrome, we have shown that *A. muciniphila* administration promotes differential and common alterations of the metabolome in individuals according to the form of the administered bacterium, i.e. the pasteurized versus the alive form.

## Results

### Pasteurized and alive A. muciniphila effects are linked to distinct and shared modulations of the serum metabolome

**Figure 1.**
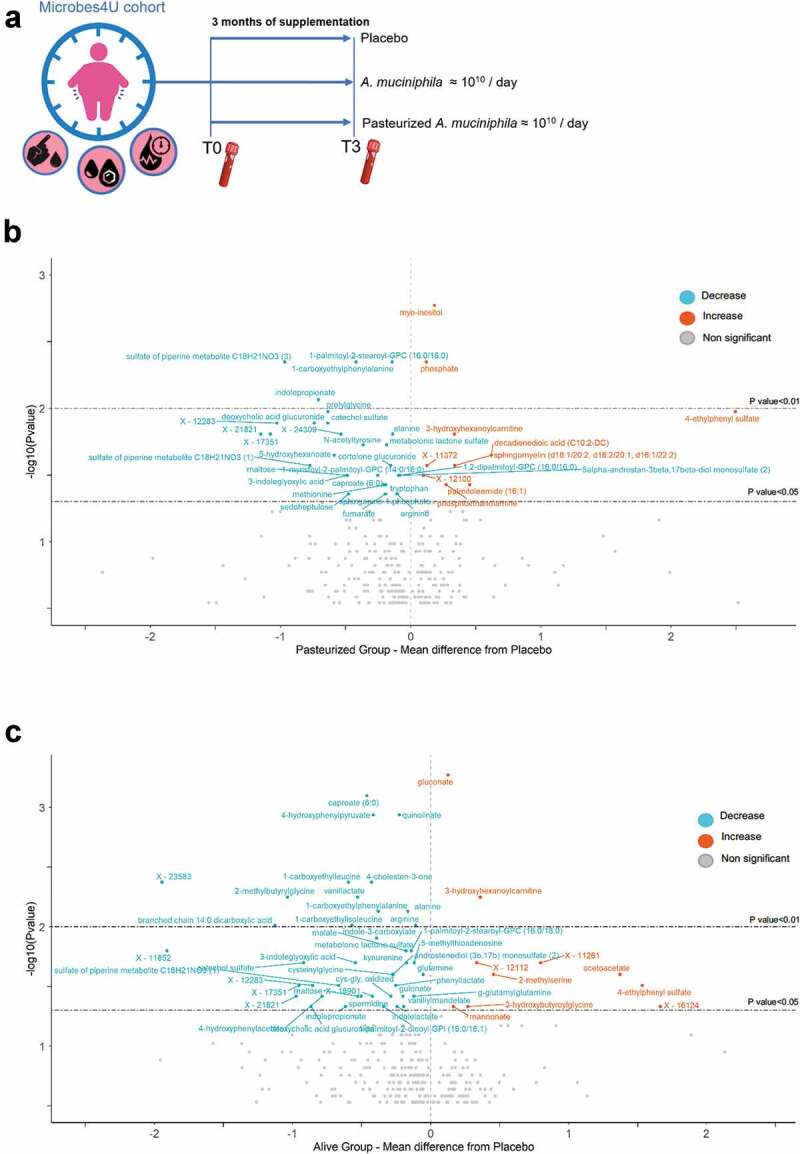
Differential and common metabolomic effects of both treatments relative to the control group. (a) Study design of the Microbes4U© intervention. 52 obese or overweight individuals diagnosed with a pre-diabetic state and a metabolic syndrome were recruited and started the intervention. After three months of supplementation, blood samples were collected from 32 individuals. (b-c) Volcano plots depicting the metabolites which most notably changed following 3 months of supplementation with pasteurized (b) or alive *A. muciniphila* (c), under comparison with the placebo interventional effect. Labeled points represent metabolites for which the results of the Mann–Whitney *U* test comparing deltas of the treatment groups versus the placebo group were significant. Metabolites marked in red, exhibiting positive value for ‘mean difference from placebo’ are compounds that increased significantly following treatment with *A. muciniphila*. Metabolites marked in blue, exhibiting negative value for ‘mean difference from placebo’ are compounds that decreased significantly following treatment with *A. muciniphila*. The compounds which did not satisfy the condition of *p* < .05 for the univariate Mann–Whitney *U* test appear in yellow, and are not labeled. For readability, metabolites whose global delta exceeded the absolute value of 4, and for which the – result of the Mann–Whitney *U* test was not significant, were omitted from the plot. Metabolites for which the – log_10_Pvalue was below 0.5 were also discarded from the plot. The two dash lines correspond to *p*-value cutoffs of 0.05 and 0.01, respectively. See also Table S1 for the detailed list of the metabolites, with corresponding value and pathway

Because this work was initiated in the continuity of what we previously published, the approach adopted for the analysis of the metabolome is similar to what has been described previously.^1,9^ Hence, for each metabolite, we measured the interventional effect per group, by calculating the mean differential value between the two main time-points, that is before and after 3 months of supplementation with the bacterium or the placebo (*i.e*., T0 and T3). The numbers obtained for both treatment groups were then subtracted from the ones of the placebo group to get the mean difference from placebo for each metabolite. We then assessed metabolites that were specifically and significantly modulated by the intervention through a comparison of differences between treatment groups and the placebo group (Figure 1). The two volcano plots displayed in Figure 1 provide an overview of the differential response between the two treatments at the level of the metabolome.

Compared to the placebo group, a total of 54 and 41 metabolites were identified as specifically and significantly modified following 3 months of supplementation with alive or pasteurized *A. muciniphila*, respectively. Briefly, 9 metabolites increased and 32 decreased in the pasteurized group, while 11 increased and 43 decreased in the alive group. The list of metabolites, with details of the corresponding pathways, is provided in Supplementary Tables 1 and 2. Of note, several metabolites related to the oxidative state were modulated. Cysteinylglycine (cys-gly) and oxidized cys-gly, two glutathione cycle intermediates, were significantly decreased by the alive from compared to the placebo group. Moreover, plasma levels of cysteine glutathione disulfide were significantly reduced in the alive group compared to baseline value (matched paired *t*-test, pv = 0.04). Global screening reveals differential modulation of the circulating metabolome between both treatment groups, with distinct signatures according to the form administrated. Nevertheless, it is worth underlining that alanine, arginine, caproate, 1-carboxyethylphenylalanine, and 3-hydroxyhexanoylcarnitine are among the metabolites that were altered in the same way regardless of the form administered. In conclusion, untargeted metabolomic profiling reveals both shared and distinct effects of alive and pasteurized forms on the serum metabolome in individuals with excess body weight and insulin resistance.

**Table 1.** Influence of *A. muciniphila* on the metabolome is closely linked to ketone body-related metabolism

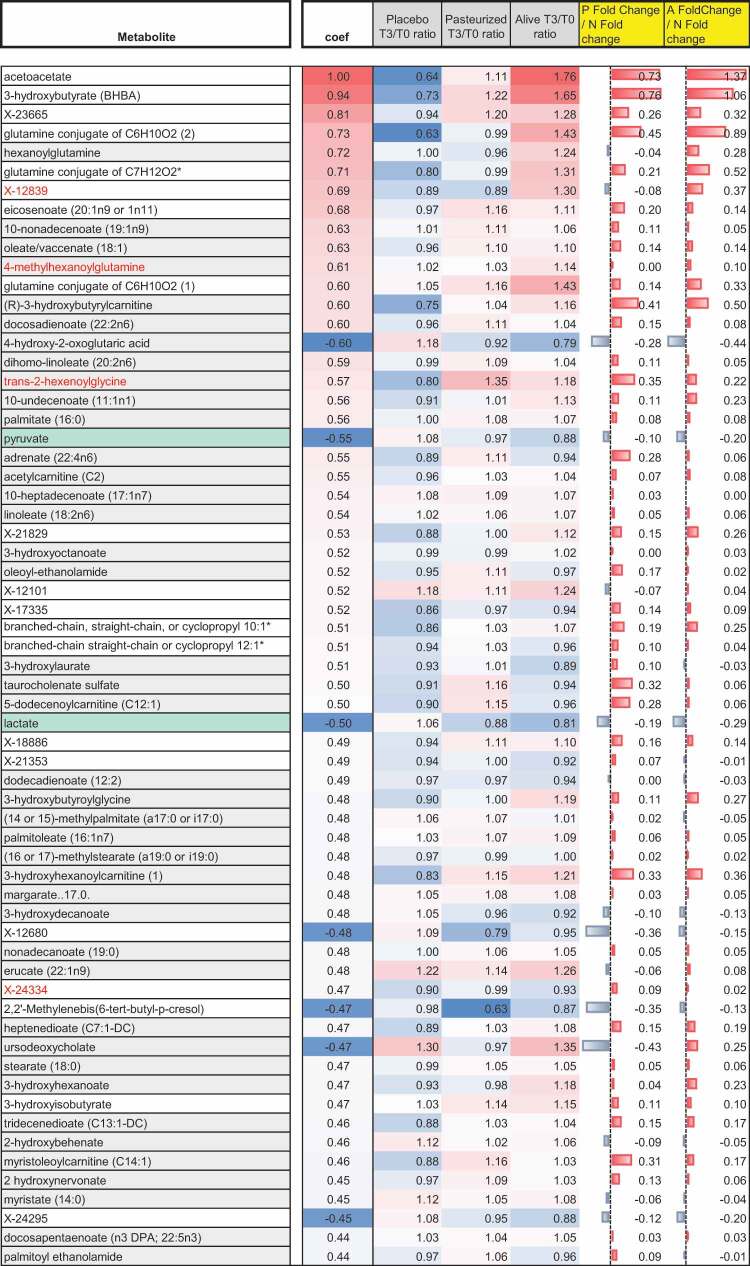

Top metabolites correlating most to acetoacetate at baseline (n = 52 biological samples). Metabolites for which the percentage of detection was below 100% percent are shown in red. A green background was used to highlight metabolites belonging to the lipid super pathway. Metabolites of the glycolytic pathway are shown on a blue background. For each metabolite, listed values for the corresponding Spearman’s coefficient of correlation, the fold change ratio, and the mean difference from placebo according to the treatment group are indicated. Adjustment for multiple testing was performed using the Benjamini-Hochberg method. Only the metabolites that significantly correlated with acetoacetate are presented (Adjusted *p* value >0.05). “T0” refers to the median-scaled baseline value, while “T3” refers to median scale final value. Abbreviations: A, Alive; N, Non-treated (Placebo); P, Pasteurized.

### *A. muciniphila* negatively modulates circulating levels of several amino acid-derived metabolites potentially associated with hepatic function

**Figure 2.**
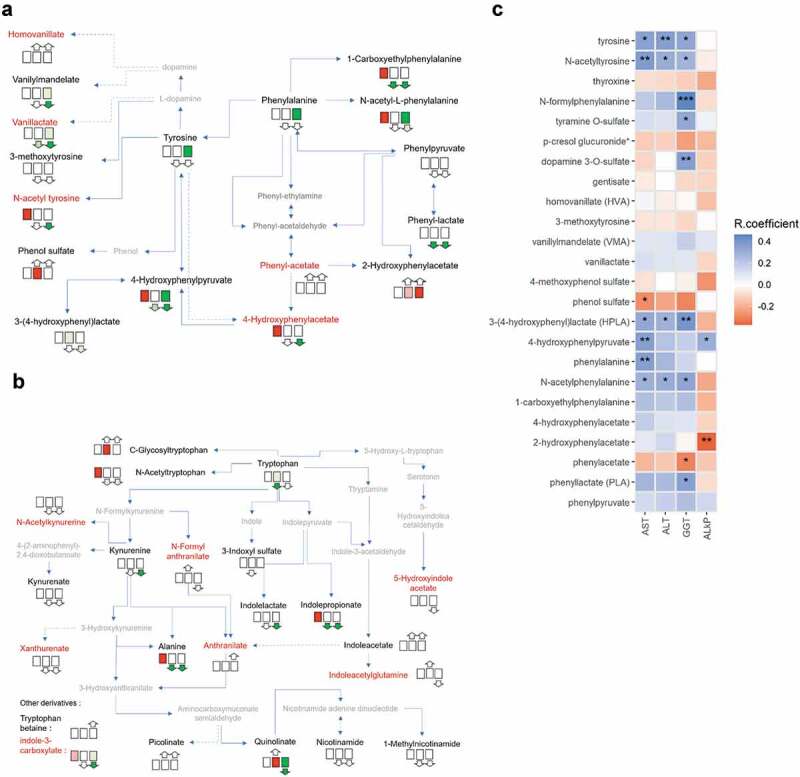
*A. muciniphila* negatively modulated circulating levels of several amino acids-derived metabolites that correlated with hepatic dysfunction Qualitative changes in phenylalanine, and tyrosine (a), and tryptophan (b) metabolites in plasma following the intervention. Intermediate metabolites which were not assayed are shown in gray and written in a smaller font. The routes including several steps and intermediaries not shown are represented by a dashed line. Enzymes are omitted. Compounds shown in red had a percentage of detection below 100% in our cohort. The trio of boxes below each metabolite represents the three groups, from left to right, given placebo, pasteurized, and alive *A. muciniphila*. Matched-pairs t-tests were performed on median scaled, log-transformed data, to verify changes from baseline (intragroup changes). Boxes with filled background mark metabolites that showed significant changes (p ≤ 0.05). The color represents the mean change from baseline value per group: red and green indicate a significant mean increase or decrease following intervention for the corresponding metabolite, respectively. Mann–Whitney *U* tests were performed to compare the differential values of both treated groups versus the placebo group (intergroup changes). Light red – and light green-shaded cells indicate 0.05 < *p*-value<0.10. The box was left emptied when the test was not significant. The direction of the arrow below both intervention groups indicates whether the metabolite had globally increased or decreased compared to the placebo effect. When the Mann–Whitney *U* test was significant (p ≤ 0.05), the arrow was colored according to the direction of the global change (red: increase, green, decrease). Light red – and light green-shaded cells indicate 0.05<* pvalue*<0.10. (c) Baseline Spearman’s correlation matrix between plasmatic hepatic enzymes and metabolites of the tyrosine and phenylalanine metabolism in the Microbes4U© cohort (n = 52). Negative correlations are colored in shades of blue and positive correlations in red. *: *p* < .05; **: *p* < .01; *** : *p* < .001

Comparison of both volcano plots led to the observation that a substantial number of metabolites belonging to the amino-acid super pathway were significantly affected by the intervention; irrespective of the form, suggesting a common path induced by both forms of the bacterium. More specifically, several intermediates of tyrosine, phenylalanine, and tryptophan metabolism arose in the negative frame of both volcano plots. Based on this observation, we laid out pathway mapping for the quantified amino acids-related intermediates, adding annotations for the effect of the intervention (Figure 2). Briefly, this mapping aimed at summarizing all outputs from statistical analysis seeking for differences between groups (placebo versus treated) and within-groups (T0 versus T3) in serum metabolome. Exploration of the tyrosine and phenylalanine pathways showed a significant increase of 4-hydroxyphenylpyruvate, 4-hydroxyphenylacetate, 1-carboxyethylphenylalanine, N-acetyl-phenylalanine, and N-acetyl tyrosine in the placebo group (Figure 2a). Conversely, most intermediates, if not significantly decreased, tended to be reduced in individuals treated with *A. muciniphila* compared to baseline value and/or to the placebo group (Figure 2a). Similarly, although the effects were mostly dominated by trends when the markers were considered individually, the analyses of the tryptophan pathways showed an overall downregulation of the serum levels of several intermediates following both interventions compared with the placebo effect (Figure 2b). Collectively, administration of *A. muciniphila* seemed to alleviate the elevation observed in the serum levels of diverse intermediates of tyrosine, phenylalanine, and tryptophan metabolism occurring over time in treatment-naïve overweight subjects with prediabetes. Because numerous elements from the literature suggest a link between elevated amounts of tyrosine and phenylalanine-derived compounds and severity of liver dysfunction,^11–14^ bivariate correlational analysis was performed on all subjects at baseline to assess relationships between serum concentration of hepatic enzymes, including transaminases, and quantified metabolites related to tyrosine and phenylalanine pathway in our cohort. The obtained Spearman’s correlation matrix indicated that both amino acids and several derived compounds were significantly and positively correlated with the serum levels of hepatic enzymes (Figure 2c). Conversely, three metabolites showed an inverse correlation with some enzymes. More specifically, phenol sulfate correlated negatively with aspartate aminotransferase (AST), phenylacetate correlated negatively with gamma-glutamyl transferase (GGT) and 2-hydroxyphenylacetate correlated negatively with alkaline phosphatase (AlKP). Interestingly, those metabolites were increased in response to the intervention while being decreased in the placebo group (Figure 2a).

### A. muciniphila-induced metabolome changes are closely linked to ketone bodies-related metabolism, including acylcarnitines

**Figure 3.**
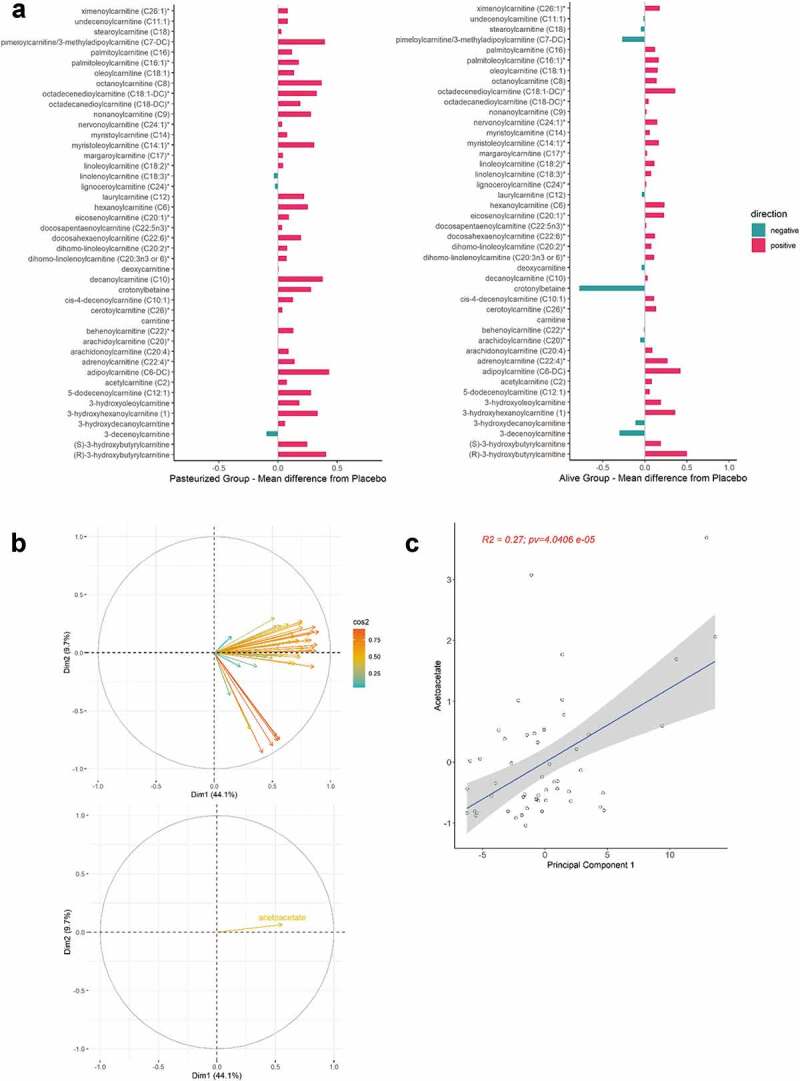
*A. muciniphila* administration was associated with a positive modulation of acylcarnitine-circulating levels. (a) Bars represent the mean difference from placebo of the relative plasma concentrations of measured acylcarnitines for the groups given pasteurized and alive *A. muciniphila*. The color indicates the direction of the global delta. (b) Principal component analysis of variables for acylcarnitines and direction of vector for acetoacetate if applied to the acylcarnitine variables plot. (c) Scatterplot showing the linear relation between first principal components and acetoacetate

Impacts of *A. muciniphila* daily administration on the serum metabolite profiles encompassed a significant increase of acetoacetate (Figure 1). Given the numerous similarities existing between the beneficial effects driven by *A. muciniphila* and ketone bodies, we further explored the relationship existing between ketone bodies and other metabolites in our cohort by performing multiple correlation analyses. Table 1 lists the top metabolites that correlated significantly with acetoacetate at baseline. The table also intends to show how the metabolome associated with ketone bodies evolved during the intervention. Perturbations observed in the listed metabolites were quite similar between both *A. muciniphila* treatments compared to placebo. Moreover, although modest for most of them, modifications were often closely proportional to the value of the correlation coefficient. In other words, when a metabolite correlated positively with acetoacetate at baseline, then this metabolite increased following treatment, regardless of the form of administration, and inversely. For instance, pyruvate and lactate, two endproducts of the glycolytic pathway, not only correlated negatively with acetoacetate at baseline but also decreased in the serum as a result of both interventions. Noteworthily, a large majority of the top metabolites belong to the lipid super pathway, and among those, many are acylcarnitines. This prompted us to look at the profile of all acylcarnitines assayed in our study. We observed that most of them increased following both treatments (Figure 3a). Higher serum levels of acylcarnitines could be a downstream consequence of enhanced fatty acid β-oxidation. Consistent with our previous correlational analysis, when performing principal component analysis (PCA), we observed that acetoacetate correlated strongly and significantly with the dimension one of the PCA that best resumes variance among acylcarnitines (Figure 3 B-C).

### A. muciniphila cross-talks with ketogenesis, lipid metabolism, and glycolysis-related metabolites

**Figure 4.**
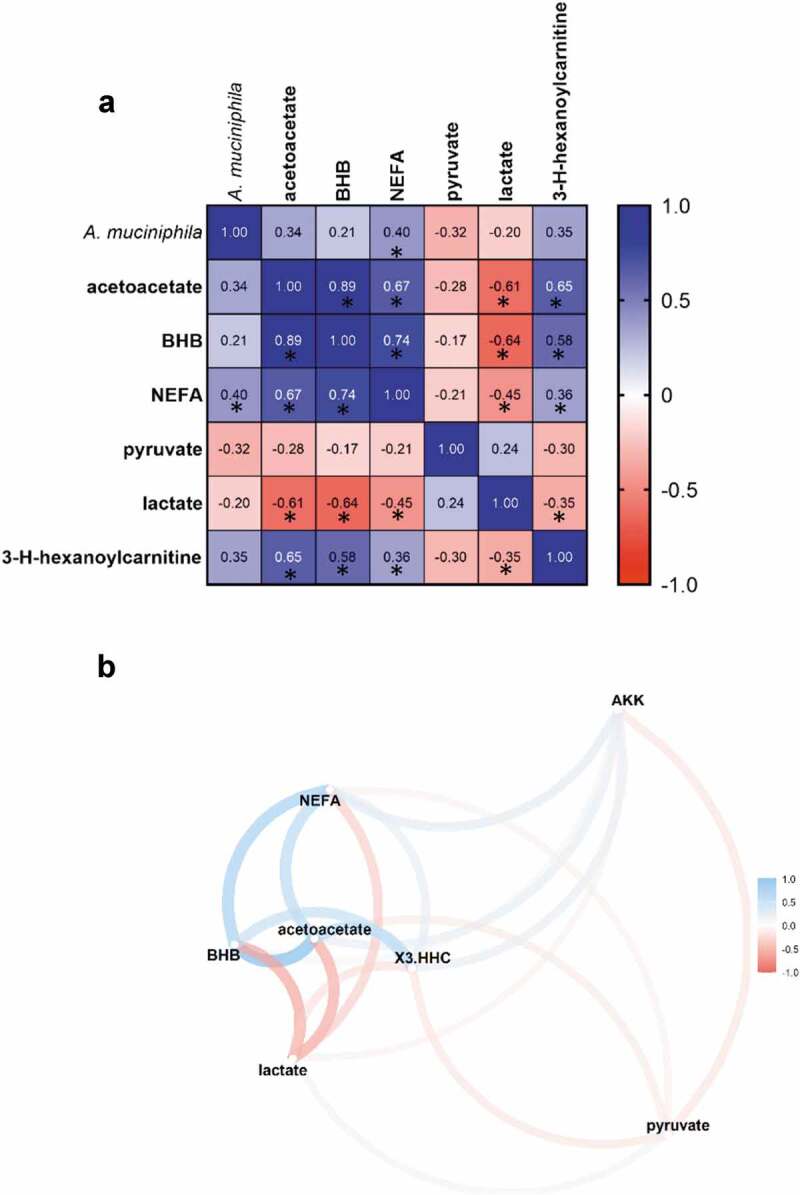
*A. muciniphila*’s cross-talk with ketogenesis, lipid metabolism and glycolysis related metabolites. (a) Spearman’s rank correlation matrix of *A. muciniphila* and plasma metabolites belonging to ketone bodies metabolism, TCA cycle, and lipid metabolism, expressed as differential value (delta) (*P < .05). (b) Correlation Network map illustrating the Spearman’s correlation matrix. Metabolites that were highly correlated are brought together. The positioning of the metabolites was calculated by multidimensional scaling of the absolute values of the correlations. Gradient color, distance, and thickness of the lines were applied to metabolites nodes depending on coefficients of correlation. Negative and positives correlations are colored in shades of red and blue, respectively. Abbreviations: Akk, *A. muciniphila*; BHB, 3-hydroxybutyrate; NEFA, non-esterified fatty acids; X3.HHC, 3-hydroxhexanoylcarnitine

To strengthen the association between ketone bodies, glycolysis, and acylcarnitines in the context of our intervention, we generated a Spearman correlation matrix, calculated between the differential value of relevant metabolites and *A. muciniphila* (Figure 4a). Since acylcarnitines represent markers of fatty acid efflux destined for β-oxidation, we also considered the plasma concentration of non-esterified fatty acids (NEFA). Figure 4b is the direct representation of the matrix in a network format. Ketone bodies correlated positively with acylcarnitine and NEFA, suggesting that ketogenesis was enhanced alongside an increased transport of lipids. This cluster of metabolites correlated negatively with lactate and pyruvate, suggesting that ketogenesis preferentially used as substrate the acetyl-CoA produced by β-oxidation rather than glycolysis. Finally, although non-significant, except for NEFA, *A. muciniphila* correlated positively with elements relative to ketogenesis and fatty acids transport, and negatively with elements belonging to the glycolysis.

## Discussion

Observations accumulated from both correlational studies in humans and preclinical studies in mice strongly positioned *A. muciniphila* as a “next-generation beneficial microbe”.^2^ While many questions remained unanswered, previous and ongoing studies sought to shed light on the mechanisms behind the promotion of healthy status by *A. muciniphila*. In the framework of the Microbes4U© study, we conducted a large prospective serum metabolomic analysis to identify novel metabolite signatures connected with the beneficial effects of 3 months daily supplementation with either alive or pasteurized *A. muciniphila* to humans in the context of metabolic syndrome.

Following the characterization of metabolomic profiles of our cohort, we conducted an intensive search through the literature for the key metabolite discriminating the placebo effect from treatment effect, to uncover its connection with human health. Myo-inositol and phosphate were both significantly increased in response to pasteurized *A. muciniphila* exposure in comparison with the placebo. Administration of myo-inositol is associated with positive impacts in term of cardiovascular risk factors and diabetes incidence, notably through improvement of insulin sensitivity, in various pathological conditions such as childhood obesity, gestational diabetes, or polycystic ovary syndrome.^15–17^ Moreover, *A. muciniphila* was strongly positively correlated with myo-inositol in a depressive rat model exhibiting reduced intestinal amount of the bacterium.^18^ Notably, two bacteria (*Dysosmobacter welbionis* and *Anaerostipes hadrus*) recently identified as potentially beneficial against obesity and diabetes and also linked to higher thermogenesis, were shown to use myo-inositol to produce butyrate or propionate.^19,20^ Regarding phosphate, two studies have described positive associations between a low phosphorus status and a higher risk for incident obesity.^21,22^ Our results showed decreased levels of α-hydroxycaproate (Sup. Table 1) and 1-palmitoyl-2-stearoyl-GPC in the group given the pasteurized *A. muciniphila*. Interestingly, α-hydroxycaproate predicted cardiovascular mortality in atherosclerotic patients,^23^ while 1-palmitoyl-2-stearoyl-GPC was found to be elevated in adipose tissues from obese individuals, in the plasma of dyslipidemic mice, and to be linked with obesity-cardiac lipotoxicity.^24–26^ Collectively, these findings echo the widely accepted anti-atherogenic and anti-obesogenic properties of the bacterium (reviewed in^27^).

Major changes in the metabolome of the participants treated with the living form included down-modulation of caproate and 2-methylbutyrylglycine. Medium-chain fatty acid caproate is well documented in the literature as positively associated with type 2 diabetes (T2D), inflammatory bowel disease (IBD), rheumatoid arthritis, or Hepatitis B virus-induced cirrhosis.^28–31^ Interestingly, one common feature for all of those conditions is a poor representation of *A. muciniphila*.^5,32,33^ In the same line, *A. muciniphila* has been suggested to exert neuroprotective effects^2,5^ and it is worth underlining that 2-methylbutyrylglycine is a metabolite described as a neurotoxin and that *A. muciniphila* treated subjects had lower levels of this metabolite.^34^ Effects of living *A. muciniphila* were also characterized by an elevation of circulating levels of gluconate and 3-hydroxyhexanoylcarnitine. Although largely described in microbiology, the function of gluconate in human physiology remains unclear.^35^ However, recent metabolomic studies highlighted a potential role in redox homeostasis through promotion of GSH recycling, in accordance with its metabolomic connection to the pentose phosphate pathway.^36^ Finally, consistent with *A. muciniphila* being a signature of longevity and being underrepresented in age-related diseases,^37,38^ 3-hydroxyhexanoylcarnitine was described as a potent predictor of healthy aging.^39^

Our analytic approach suggested some divergences in the mode of actions between living and pasteurized *A. muciniphila*. However, the metabolomics response triggered by both forms shared some striking similarities, such as a down-regulation of tyrosine, phenylalanine, and tryptophan metabolism. Based on these findings, a recent metagenome-wide association study showed that *A. muciniphila* was negatively associated with circulating levels of phenylalanine in a human cohort comprising lean and obese individuals.^40^ In the same study, a large number of microbial species overrepresented in obese individuals were positively correlated with tyrosine. Noteworthily, as indicated by several human studies, tryptophan, tyrosine, and phenylalanine metabolisms are connected positively with body mass index, metabolic syndrome score, and T2D risk.^41–44^ Some reports even underscored strong links between an altered tyrosine or phenylalanine metabolism and premalignant liver diseases, acute or chronic liver failure, and related-mortality.^11–14^ More importantly, these observations were linked to the gut microbiota. Along with this, we further speculate that alterations in the aforementioned amino acids metabolism might be linked to the hepatoprotective effect of the bacterium. Spearman correlation analysis confirmed the existence of a global link between biomarkers of hepatic dysfunction and relative elevations of intermediates of tyrosine and phenylalanine metabolism. These findings reflect our previous observations of significantly reduced levels of transaminases and gamma glutamyl transferase in participants from the same cohort treated with the pasteurized *A. muciniphila*.^9^ Of note, bariatric surgery, which leads to sustained weight loss, improved glycemic control and a bloom in gut *A. muciniphila*,^40,45–47^ also results in down-modulation of tryptophan and intermediates of the pathway, especially the kynurenine pathway, which in our study was attenuated.^48^ This being said, one exception relates to indolepropionate, a gut-derived product of tryptophan metabolism and a largely recognized health-associated metabolite, the reduced blood level of which constitutes a hallmark of various metabolic diseases.^49,50^ In our studies, the serum of participants treated with pasteurized *A. muciniphila* showed reduced levels of indolepropionate. Whether this feature is unbeneficial is a relevant question, but given that all the other metabolites are pointing in another direction, this finding warrants further investigation.

Tyrosine, tryptophan, and phenylalanine are amino acid required in gut protein putrefaction, a process that is associated with disruption of gut homeostasis,^51^ which is affected by bariatric surgery.^52,53^ Thus, our result could potentially reflect a decrease of protein putrefaction. Except for hydroxyphenylacetate, typical putrefaction-derived metabolites were not significantly modulated by the intervention (*i.e*. putrescine, *p*-cresol, phenylacetylglutamine, spermidine).^54^ However, that our results might related on modulation of gut protein or putrefaction remains suppositional without fecal analysis, since blood metabolome does not always reflect colon luminal metabolome.

Our data indicated significant reduction of alanine and arginine irrespective of the form of the administered *A. muciniphila*. Both amino acids were shown to be highly correlated with insulin resistance in a cohort of obese children.^55^ Connections of these amino acids to metabolic unwellness were further confirmed in other human studies, including a recent systemic review of human studies addressing metabolomic profiling of obesity.^43,44,56,57^

Despite being a heterogeneous group of compounds, acylcarnitines were markedly increased in the serum of both treatment groups. Levels of most acylcarnitines naturally rise in the peripheral blood upon fasting condition or following exercise, as a reflect of increased transport of fatty acids, and subsequent increase in fatty acid β-oxidation rate.^58,59^ Thereby, our observations are likely to be due to increased mitochondrial efficiency for fatty acid β-oxidation. In line with this, a recent in vivo study in mice confirmed that *A. muciniphila* inoculation not only promoted efflux of fatty acids through positive modulation of genes involved in hepatic lipid transport, but also resulted in a higher number of mitochondria.^60^ Our hypothesis is further substantiated by recent research, including studies performed in our lab, showing that direct inoculation or indirect diet-mediated *A. muciniphila* enrichment in rodents activates a network of genes involved in lipid oxidation in adipose and liver tissue.^3,60–67^ These include gene encoding for PPARα, peroxisome proliferator-activated receptor-gamma coactivator-1 alpha (PGC1α), and carnitine palmitoyl‐transferase I (CPT‐I). PPARα is a key sensor of fatty acid flux whose activation, together with PGC1α, stimulated the expression of CPT-I isoform α.^68^ This enzyme catalyzes the formation of acylcarnitines by condensation of activated fatty acids (acyl‐CoAs) to carnitine, thus regulating the entry of acyl‐CoAs into the mitochondrial matrix.^69^ Of note, we have recently shown in the Microbes4U© cohorts that *A. muciniphila* supplementation induced an increase blood level of 2-palmitoyl glycerol, a newly identify PPARα agonist.^1^

A metabolic switch toward the use of fatty acid as the major energy substrate was suggested to potentially result in higher oxygen consumption and energy expenditure.^70^ Of note, we have previously described that administration of pasteurized *A. muciniphila* in a diet-induced obesity model led to increased energy expenditure, oxygen consumption, and carbon dioxide production in a thermogenesis-independent manner.^71^

If not oxidized in the TCA cycle, as suggested by reduced amount of fumarate and malate, an alternative route for β-oxidation-derived Acetyl-coA is ketogenesis. Our results indicated a significant increase of acetoacetate in the group given live *A. muciniphila*. We also noted a similar trend for 3-hydroxybutyrate. Of note, both ketone bodies tend to increase in the group given pasteurized *A. muciniphila*. Finally, we demonstrated that the metabolites closely associated with ketone bodies shifted toward the direction of that correlation upon both interventions. Recent literature tends to emphasize the key role of ketone bodies as signaling molecules (for review see^72^). Ketogenesis can be induced by the consumption of high-fat, low-carbohydrate diets.^73^ The so-called ketogenic diet is currently getting popularity as a new efficient approach for obesity management since it promotes weight loss, glycemic control, reduced systemic inflammation among other effects.^74^ Positive effects were also well described in the context of epilepsy,^75^ a condition associated with an altered gut microbiota.^76^ Moreover, colonization with *A. muciniphila* exerts anti-seizure effects in a mice model.^77^ Adding to the physiological importance of the pathway, impaired ketogenesis was shown to exert a strong impact on steatosis development and to limit hepatic gluconeogenesis in humans.^41^ As for our result regarding amino-acid metabolism, this is also in line with numerous murine studies showing that *A. muciniphila* lowers hepatic inflammation and steatosis.^60,78–80^

This proposed mechanism of enhanced ketogenesis is supported by several lines of evidence. Indeed, Crohn’s disease, ulcerative colitis, multiple sclerosis, Alzheimer’s disease, and autism are among pathological conditions for which ketogenic diet-based therapies were reported as beneficial.^81^ Interestingly, those are conditions in which intestinal *A. muciniphila* is underrepresented, but also those in which *A. muciniphila* administration in murine models improves the phenotype,^82^ as reviewed in.^5,8,79^

On top of this, ketone bodies and *A. muciniphila* share common features in the context of healthy-aging and cancer prevention. This fact was notably highlighted in a recent metabolomic study carried out in mice receiving alive or pasteurized *A. muciniphila*. The authors described an elevation of intestinal and circulating levels of the ketone body 2β-hydroxybutyrate, and linked this to the anti-aging and anti-cancer effect of *A. muciniphila*.^83^ Along the same line, colonization of germ-free mice with *A. muciniphila* led to significant modulation of genes related to ketone body metabolism in the ileum.^84^ Finally, one study also showed increased plasma acetoacetate in chow-fed mice treated with live *A. muciniphila*.^85^

Consistent with their implications in the maintenance of cellular redox homeostasis, ketone bodies, together with the gluconate metabolite, might be partially responsible for the observed lower levels of indirect markers of oxidative stress (*i.e*. cys-gly, oxidized cys-gly, cysteine glutathione disulfide). Favorable redox state, in turn, preserves the function of the carnitine/acylcarnitine carrier involved in entry of fatty acids in the mitochondria.^86^

**Figure 5.**
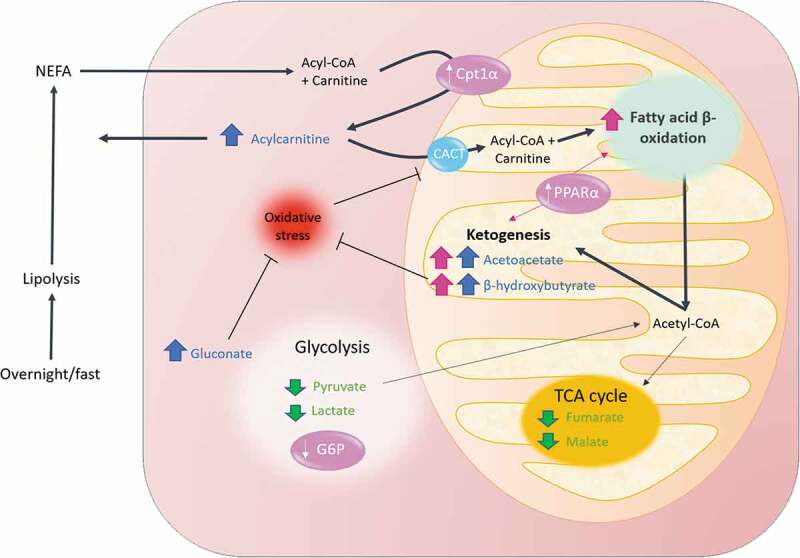
Overview of the mechanism proposed for the *A. muciniphila*-mediated metabolomic switch toward β-oxidation and ketogenesis. In physiological conditions, overnight fast is associated with high lipolytic rates to increase the availability of substrates for β – oxidation. This results in a substantial efflux of non-esterified fatty acids and esterified carnitine in the plasma. Entry of acyl-CoA into the mitochondria is facilitated by the acylcarnitine transport system, while part of the generated acylcarnitines are released in the plasma. Fatty acids are then broken down into Acetyl-coA through β-oxidation in the mitochondria. Administration of *A. muciniphila* in insulin-resistant overweight individual turndowned glycolysis and amplified the aforementioned pathway and triggered a switch toward the use of Acetyl-CoA for ketogenesis rather than toward the TCA cycle. In turn, elevated levels of ketones bodies, alongside gluconate, may contribute to reduce the redox state, favoring the activity of CACT. The thicker the arrow the stronger the corresponding pathway was triggered. Elements and arrows in pink reflect known facts from literature relative to *A. muciniphila* action. Increase and decrease metabolites are shown in blue and green, respectively. CACT, Carnitine-acylcarnitine translocase; CPT1α, carnitine palmitoyl‐transferase I; G6P, Glucose-6-phosphatase; PPARα, peroxisome proliferator-activated receptor alpha; TCA, tricarboxylic acid cycle

Our assumptions need further validation in larger prospective human studies. However, when all the observations described in this paper are grouped, a metabolic pattern emerges. A proposed metabolic mechanism for the plasmatic metabolome associated with *A. muciniphila* exposure is displayed in Figure 5.

Briefly, our data raise the hypothesis that administration of *A. muciniphila* in prediabetic subjects with metabolic syndrome enhances hepatic fatty acids delivery and subsequent β-oxidation, but attenuates acetyl-CoA oxidation in the TCA cycle, leading preferentially to ketogenesis. Downstream metabolomic effects of *A. muciniphila* also lead to downregulation of the metabolism of several amino acids, whose implications in various metabolic disorders are convincingly demonstrated in the scientific literature. We notably identified a global downregulation of the tyrosine and phenylalanine metabolism as a potential mechanism contributing to the hepato-protective effects of *A. muciniphila*. Our analysis also revealed specific modulations of metabolites with emerging implications in health and diseases, including disorders for which *A. muciniphila* was shown to be negatively associated with. To close the discussion, it is noteworthy to raise the question of whether or not our observations are the cause or the consequence of the improvement of the metabolic state, constituting thus a limitation of our study. Although we acknowledge the modest scope of each observation taken individually, the strength of our study lies in the coherence between the global scheme that we propose, born from the junction between all observations, and the existing literature in relation to *A. muciniphila*. Those new insights bridge the recognized links between obesity-associated metabolomic hallmarks (including oxidative stress, alteration of amino acids metabolism, markers of hepatic disorders) and isolated observations connecting *A. muciniphila* to its health-promoting status.

## Materials and methods

### Participants and study design

The Microbes4U© study used a randomized, double-blinded, parallel, placebo-controlled design. The cohort consisted of overweight or obese individuals (body mass index (BMI)>25 kg/m2), aged between 18 and 70 years. Subjects were eligible for the study if they present with a newly diagnosed pre-diabetic state and a metabolic syndrome. Pre-diabetes was evaluated using HOMA-modeling of insulin sensitivity (HOMA Calculator, University of Oxford), with insulin resistance considered if participant’s insulin sensitivity was below 75%. The diagnosis of a metabolic syndrome was established according to the National Cholesterol Education Program Adult Treatment Panel III definition, that is, at least three of the five following criteria: fasting glycemia >100 mg dL-1; blood pressure >130/85 mmHg or antihypertensive treatment; fasting triglyceridemia >150 mg dL-1; high-density lipoprotein (HDL) cholesterol <40 mg dL-1 for men, <50 mg dL-1 for women; and/or waist circumference >102 cm for men, >88 cm for women. Exclusion criteria and details regarding study design were previously extensively described.^9^ Briefly, between 2015 and 2018, 52 volunteers were enrolled at the Cliniques Universitaires Saint-Luc in Belgium and started the intervention. Following screening, participants were randomized into four parallels groups, receiving for 12 weeks either a sterile liquid solution of PBS-containing glycerol as a placebo, or an equivalent volume of the same solution plus 10^10^ alive *A. muciniphila*, 10^9^ alive *A. muciniphila*, or 10^10^ pasteurized *A. muciniphila*. Pasteurization consisted of heat treatment at 70°C for 30 min of fresh *A. muciniphila*. Participants and physicians were both blinded to treatment allocation. Blood samples were collected in the fasting state at the beginning of the intervention prior to any administration and on the last day of the trial. The study protocol was approved by the local ethical committee on July 2015 (comité d’éthique hospitalo-facultaire UCLouvain, Cliniques Universitaires Saint-Luc) under the number 2015/02JUL/369, and the study was registered at clinicaltrial.gov under the number NCT02637115.

This study aimed at further extending our preliminary analysis, beyond safety, tolerability, and feasibility assessment.^8,9^ Aiming will to continue the effort undertaken, on the one hand, in the characterization of the effects and, on the other hand, in the exploration of the underlying biological mechanisms, our metabolomic analysis focused on the comparison of the control group with the groups treated with the highest dose tested, that is 10^10^ bacteria per day. Accordingly, 32 subjects completed the trial after 3 months of daily supplementation. Baseline characteristics of that cohort were previously described.^9^

### Blood Serum biochemical analysis

Plasma samples were collected after overnight fasting (8 hours minimum) in lithium-heparin coated tubes. One set of tubes was sent directly to the hospital laboratory for several blood analyses including liver enzymes activities. The remaining samples were transported on ice from the sampling center to the research laboratory. Plasma was immediately isolated from whole blood by centrifugation at 4200 g for 10 min at 4°C and stored at −80°C. For metabolomics profiling, 100 μl of plasma was aliquoted and transported on dry ice to Metabolon Inc.

Alkaline phosphatase (AlkP) and gamma-glutamyl transferase (GGT) were assayed by enzymatic colorimetric method (Cobas 8000 – Roche Diagnostics), while aspartate aminotransferase (AST) and alanine aminotransferase (ALT) were assayed by enzymatic methods (International Federation of Clinical Chemistry and Laboratory Medicine) without activation by pyridoxal phosphate (Cobas 8000 – Roche Diagnostics). Plasma non-esterified fatty acids (NEFAs) were measured using kits coupling an enzymatic reaction with spectrophotometric detection of the reaction endproducts (Diasys Diagnostic and Systems, Holzheim, Germany) according to the manufacturer’s instructions.

### Untargeted metabolomics assays

Untargeted metabolomics profiling using high-resolution mass spectrometry with hydrophilic interaction chromatography was applied to all samples via Metabolon’s HD4 multi-platform techniques. The non-targeted mass spectrometry analysis of Metabolon Inc. (North Carolina, USA) was previously described.^87^ Briefly, it comprised ultra-performance liquid chromatography/mass spectrometry with a heated electrospray ionization source and mass analyzer. Following proper handling, samples were first prepared using the automated MicroLab STAR® system from Hamilton Company. The resulting extract was divided into several fractions, analyzed by four ultra-high-performance liquid chromatography-tandem mass spectrometry according to the Metabolon pipeline. Biochemical identification of metabolites contained in one sample was then performed by comparison to a reference library of purified standards consisting of more than 33000 metabolites. The comparison was based on retention time/index, mass-to-charge ratio (m/z), and chromatographic data (MS/MS spectral data) using software developed at Metabolon. Further details regarding quality controls, data extraction, curation, quantification, and bioinformatics were previously described.^88^

### Statistical analysis

Metabolomic profiling detected a total of 1169 compounds, 947 of which were of known identity, while 222 were of unknown identity (X-number). Because of a very weak detection rate, metabolites related to drugs (*i.e*. analgesic, neurological, respiratory, antibiotic, psychoactive) were not considered in the analysis. Prior to formal statistical analysis, each compound was scaled to the median value in order for the median value for each metabolite to equal one. Missing data were imputed with the minimum observed value for that compound. Evaluation of the interventional effect within each group was assessed using Matched paired T-test on log-transformed, median-scaled data (intragroup change). The ratio or fold change was calculated for each metabolite by dividing the final value by the baseline value, to evaluate the mean average individual changes between both timings, according to the following formula:

$$Fold\;Change_{group}=Mean_{group}{\left(T3value/T0value\right)}_{ind}$$

where ‘ind’ referred to each participant and ‘group’ to the group.

Similarly, the mean change from baseline to end of treatment was then calculated for each metabolite, within each group, by subtracting the value obtained at Time 0 from the value obtained at Time 3 months for each participant, according to the following formula:

$$Mean\;difference_{group}=Mean_{group}{\left(T3value-T0value\right)}_{ind}$$

By subtracting the mean difference, thus calculated for each treatment group, to the one calculated for the placebo group, we obtained the “mean difference from placebo” for each metabolite, according to the following formula:

$$\begin{array}{r} \;\;\;Mean\;difference\;from\;placebo\\ =Mean_{group}\left[{\left(T3value-T0value\right)}_{ind}-Mean\;difference_{placebo}\right] \end{array}$$

This value gave a global measurement of the interventional effect when compared to the evolution of the placebo group. Mann–Whitney *U* tests were then performed to compare whether the mean difference obtained for the two treatment groups was significantly different from the one calculated for the placebo group. Intergroup statistical analyses were conducted using SPSS v.27.0 (IBM Corporation). All tests were two-tailed and significance was set at *p* < .05.

Results from those extended univariate analyses were collected and presented using two volcano plots, one per treatment group. X-axis and Y-axis represent the mean difference from the placebo, and statistical significance as the negative log of Mann–Whitney *P*-values, respectively. The significantly increased or decreased metabolites are labeled in red or blue, respectively, while non-significant genes are shown as color dots without labeling.

Spearman’s correlation of acetoacetate against all others metabolites of the dataset was computed on Rstudio using the package ‘Hmisc’ (version 4.5–0) with multiple testing correction via FDR estimation according to the Benjamini and Hochberg procedure. The metabolites that significantly correlated with acetoacetate and with the highest absolute coefficient of correlation were extracted and listed in a table format.

Principal component analyses were performed on Rstudio (R version 3.6.3, Rstudio Team, Boston, MA, USA) using factoextra (version 1.0.7) and factoMiner (version 2.3) packages. Linear regression was then built between the metabolite acetoacetate and the first principal component summarizing the highest variance in acylcarnitine, as previously described.^89^

The Spearman’s correlation matrix related to ketone bodies was drawn with Prism software v.9.0 (GraphPad Software). All the other plots (*i.e*. Volcano plots, Spearman’s correlation matrix, Barplots, the correlative network plot, and the Scatterplot) were constructed on Rstudio (R version 3.6.3, Rstudio Team, Boston, MA, USA) using the R packages “ggplot2” (version 3.3.2), “tidyverse” (version 1.3.0) and “corrr”(version 0.4.3).

## Supplementary Material

Supplemental Material

## References

[1] Depommier C, Vitale RM, Iannotti FA, Silvestri C, Flamand N, Druart C, Everard A, Pelicaean R, Maiter D, Thissen JP, et al. Beneficial effects of akkermansia muciniphila are not associated with major changes in the circulating endocannabinoidome but linked to higher mono-palmitoyl-glycerol levels as new PPARα agonists. Cells. 2021;10(1):185. PMID:. doi:10.3390/cells10010185. 33477821 PMC7832901

[2] Cani PD. Human gut microbiome: hopes, threats and promises. Gut. 2018;67(9):1716–19. PMID: 29934437. doi:10.1136/gutjnl-2018-316723. 29934437 PMC6109275

[3] Everard A, Belzer C, Geurts L, Ouwerkerk JP, Druart C, Bindels LB, Guiot Y, Derrien M, Muccioli GG, Delzenne NM et al. Cross-talk between Akkermansia muciniphila and intestinal epithelium controls diet-induced obesity. Proc Natl Acad Sci U S A. 2013;110(22):9066–9071. PMID: 23671105. doi:10.1073/pnas.1219451110. 23671105 PMC3670398

[4] Corb Aron RA, Abid A, Vesa CM, Nechifor AC, Behl T, Ghitea TC, Munteanu MA, Fratila O, Andronie-Cioara FL, Toma MM et al. Recognizing the benefits of pre-/probiotics in metabolic syndrome and type 2 diabetes mellitus considering the influence of akkermansia muciniphila as a key gut bacterium. Microorganisms. 2021;9. PMID: 33802777. doi:10.3390/microorganisms9030618. PMC8002498 33802777

[5] Cheng D, Xie MZ. A review of a potential and promising probiotic candidate—Akkermansia muciniphila. J Appl Microbiol. 2021;130(6):1813–1822. PMID: 33113228. doi:10.1111/jam.14911. 33113228

[6] Abuqwider J, Mauriello G, Altamimi M. Akkermansia muciniphila, a new generation of beneficial microbiota in modulating obesity: a systematic review. Microorganisms. 2021;9(5):1098. PMID. doi:10.3390/microorganisms9051098. 34065217 PMC8161007

[7] Xu Y, Wang N, Tan HY, Li S, Zhang C, Feng Y. Function of akkermansia muciniphila in obesity: Interactions with lipid metabolism, immune response and gut systems. Front Microbiol. 2020;11:219. PMID: 32153527. doi:10.3389/fmicb.2020.00219. 32153527 PMC7046546

[8] Plovier H, Everard A, Druart C, Depommier C, Van Hul M, Geurts L, Chilloux J, Ottman N, Duparc T, Lichtenstein L, et al. A purified membrane protein from Akkermansia muciniphila or the pasteurized bacterium improves metabolism in obese and diabetic mice. Nat Med. 2017;23(1):107–113. PMID: 27892954. doi:10.1038/nm.4236. 27892954

[9] Depommier C, Everard A, Druart C, Plovier H, Van Hul M, Vieira-Silva S, Falony G, Raes J, Maiter D, Delzenne NM, et al. Supplementation with Akkermansia muciniphila in overweight and obese human volunteers: a proof-of-concept exploratory study. Nat Med. 2019;25(7):1096–1103. PMID: 31263284. doi:10.1038/s41591-019-0495-2. 31263284 PMC6699990

[10] Druart C, Plovier H, Van Hul M, Brient A, Phipps K, De Vos W, Cani PD. Toxicological safety evaluation of pasteurized Akkermansia muciniphila. Journal of Applied Toxicology. 2020;41. PMID:. doi:10.1002/jat.4044. PMC7818173 32725676

[11] Jin R, Banton S, Tran VT, Konomi JV, Li S, Jones DP, Vos MB. Amino acid metabolism is altered in adolescents with nonalcoholic fatty liver disease - an untargeted, high resolution metabolomics study. J Pediatr. 2016;172:14–9.e5. PMID:. doi:10.1016/j.jpeds.2016.01.026. 26858195 PMC5321134

[12] Beyoğlu D, Idle JR. Metabolomic and lipidomic biomarkers for premalignant liver disease diagnosis and therapy. Metabolites. 2020;10(2):50. PMID:. doi:10.3390/metabo10020050. PMC7074571 32012846

[13] Bajaj JS, Reddy KR, O’Leary JG, Vargas HE, Lai JC, Kamath PS, Tandon P, Wong F, Subramanian RM, Thuluvath P. Serum levels of metabolites produced by intestinal microbes and lipid moieties independently associated with acute-on-chronic liver failure and death in patients with cirrhosis. Gastroenterology. 2020;159(5):1715–30.e12. PMID:. doi:10.1053/j.gastro.2020.07.019. 32687928 PMC7680282

[14] Caussy C, Hsu C, Lo MT, Liu A, Bettencourt R, Ajmera VH, Bassirian S, Hooker J, Sy E, Richards L. Link between gut-microbiome derived metabolite and shared gene-effects with hepatic steatosis and fibrosis in. NAFLD Hepatology. 2018;68(3):918–932. PMID: 29572891. doi:10.1002/hep.29892. 29572891 PMC6151296

[15] Mancini M, Andreassi A, Salvioni M, Pelliccione F, Mantellassi G, Banderali G. Myoinositol and D-chiro inositol in improving insulin resistance in obese male children: preliminary data. Int J Endocrinol. 2016;2016:8720342. PMID: 27882052. doi:10.1155/2016/8720342. 27882052 PMC5108849

[16] Minozzi M, Nordio M, Pajalich R. The Combined therapy myo-inositol plus D-Chiro-inositol, in a physiological ratio, reduces the cardiovascular risk by improving the lipid profile in PCOS patients. Eur Rev Med Pharmacol Sci. 2013;17:537–540. PMID: 23467955. 23467955

[17] Asimakopoulos G, Pergialiotis V, Anastasiou E, Antsaklis P, Theodora M, Vogiatzi E, Kallergi A, Sindos M, Loutradis D, Daskalakis G. Effect of dietary myo-inositol supplementation on the insulin resistance and the prevention of gestational diabetes mellitus: study protocol for a randomized controlled trial. Trials. 2020;21(1):633. PMID:. doi:10.1186/s13063-020-04561-2. 32646482 PMC7346495

[18] Song J, Ma W, Gu X, Zhao L, Jiang J, Xu Y, Zhang L, Zhou M, Yang L. Metabolomic signatures and microbial community profiling of depressive rat model induced by adrenocorticotrophic hormone. J Transl Med. 2019;17(1):224. PMID: 31307473. doi:10.1186/s12967-019-1970-8. 31307473 PMC6631535

[19] Le Roy T, Moens de Hase E, Van Hul M, Paquot A, Pelicaen R, Régnier M, et al. Dysosmobacter welbionis is a newly isolated human commensal bacterium preventing diet-induced obesity and metabolic disorders in mice. Gut. 2021;gutjnl-2020-323778. gutjnl-2020-323778. PMID:. doi: 10.1136/gutjnl-2020-323778. PMC8862106 34108237

[20] Zeevi D, Korem T, Godneva A, Bar N, Kurilshikov A, Lotan-Pompan M, Weinberger A, Fu J, Wijmenga C, Zhernakova A. Structural variation in the gut microbiome associates with host health. Nature. 2019;568(7750):43–48. PMID:. doi:10.1038/s41586-019-1065-y. 30918406

[21] Obeid OA. Low phosphorus status might contribute to the onset of obesity. Obes Rev. 2013;14(8):659–664. PMID: 23679666. doi:10.1111/obr.12039. 23679666

[22] Zhukouskaya VV, Rothenbuhler A, Colao A, Di Somma C, Kamenický P, Trabado S, Prié D, Audrain C, Barosi A, Kyheng C, et al. Increased prevalence of overweight and obesity in children with X-linked hypophosphatemia. Endocr Connect. 2020;9(2):144–153. PMID: 31910157. doi:10.1530/ec-19-0481. 31910157 PMC6993252

[23] Cardellini M, Ballanti M, Davato F, Cardolini I, Guglielmi V, Rizza S, Pecchioli C, Casagrande V, Mavilio M, Porzio O et al. 2-hydroxycaproate predicts cardiovascular mortality in patients with atherosclerotic disease. Atherosclerosis. 2018;277:179–185. PMID: 29958653. doi:10.1016/j.atherosclerosis.2018.06.014. 29958653

[24] Candi E, Tesauro M, Cardillo C, Lena AM, Schinzari F, Rodia G, Sica G, Gentileschi P, Rovella V, Annicchiarico-Petruzzelli M., et al. Metabolic profiling of visceral adipose tissue from obese subjects with or without metabolic syndrome. Biochem J. 2018;475(5):1019–1035. PMID: 29437994. doi:10.1042/bcj20170604. 29437994

[25] Shon JC, Kim WC, Ryu R, Wu Z, Seo J-S, Choi M-S, Liu, K-H. Plasma lipidomics reveals insights into anti-obesity effect of chrysanthemum morifolium ramat leaves and its constituent luteolin in high-fat diet-induced dyslipidemic mice. Nutrients. 2020;12(10):2973. PMID:. doi:10.3390/nu12102973. PMC7650530 33003339

[26] Marín-Royo G, Gallardo I, Martínez-Martínez E, Gutiérrez B, Jurado-López R, López-Andrés N, Gutiérrez-Tenorio J, Rial E, Bartolomé M, Nieto, ML et al. Inhibition of galectin-3 ameliorates the consequences of cardiac lipotoxicity in a rat model of diet-induced obesity. Dis Model Mech. 2018;11:dmm032086. PMID: 29361517. doi:10.1242/dmm.032086. 29361517 PMC5894945

[27] Verhoog S, Taneri P, Diaz ZM, Marques-Vidal P, Troup J, Bally L, Franco O, Glisic M, Muka T. Dietary factors and modulation of bacteria strains of akkermansia muciniphila and faecalibacterium prausnitzii: a systematic review. Nutrients. 2019;11(7):1565. PMID:. doi:10.3390/nu11071565. PMC6683038 31336737

[28] Al-Sulaiti H, Diboun I, Banu S, Al-Emadi M, Amani P, Harvey TM, Dömling AS, Latiff A, Elrayess MA. Triglyceride profiling in adipose tissues from obese insulin sensitive, insulin resistant and type 2 diabetes mellitus individuals. J Transl Med. 2018;16(1):175. PMID:. doi:10.1186/s12967-018-1548-x. 29940972 PMC6019324

[29] Zhang WX, Zhang Y, Qin G, Li KM, Wei W, Li SY, Yao SK. Altered profiles of fecal metabolites correlate with visceral hypersensitivity and may contribute to symptom severity of diarrhea-predominant irritable bowel syndrome. World J Gastroenterol. 2019;25(43):6416–6429. PMID: 31798278. doi:10.3748/wjg.v25.i43.6416. 31798278 PMC6881512

[30] Takahashi S, Saegusa J, Onishi A, Morinobu A. Biomarkers identified by serum metabolomic analysis to predict biologic treatment response in rheumatoid arthritis patients. Rheumatology. 2019;58(12):2153–2161. PMID:. doi:10.1093/rheumatology/kez199. 31143951

[31] Yu M, Zhu Y, Cong Q, Wu C. Metabonomics research progress on liver diseases. Can J Gastroenterol Hepatol. 2017;2017:8467192. PMID: 28321390. doi:10.1155/2017/8467192. 28321390 PMC5339575

[32] Li X, Wu S, Du Y, Yang L, Li Y, Hong B. Entecavir therapy reverses gut microbiota dysbiosis induced by hepatitis B virus infection in a mouse model. Int J Antimicrob Agents. 2020;56(1):106000. PMID: 32360229. doi:10.1016/j.ijantimicag.2020.106000. 32360229

[33] Shi N, Zhang S, Silverman G, Li M, Cai J, Niu H. Protective effect of hydroxychloroquine on rheumatoid arthritis-associated atherosclerosis. Animal Models and Experimental Medicine. 2019;2(2):98–106. PMID:. doi:10.1002/ame2.12065. 31392302 PMC6600633

[34] Knebel LA, Zanatta Â, Tonin AM, Grings M, Alvorcem L, Wajner M, Leipnitz G. 2-Methylbutyrylglycine induces lipid oxidative damage and decreases the antioxidant defenses in rat brain. Brain Res. 2012;1478:74–82. PMID:. doi:10.1016/j.brainres.2012.08.039. 22967964

[35] Rohatgi N, Nielsen T, Bjørn S, Axelsson Í, Paglia G, Voldborg B, Palsson B, Rolfsson Ó. Biochemical characterization of human gluconokinase and the proposed metabolic impact of gluconic acid as determined by constraint based metabolic network analysis. PLoS ONE. 2014;9(6):e98760. PMID:. doi:10.1371/journal.pone.0098760. 24896608 PMC4045858

[36] Chen Y, Golla S, Garcia-Milian R, Thompson DC, Gonzalez FJ, Vasiliou V. Hepatic metabolic adaptation in a murine model of glutathione deficiency. Chem Biol Interact. 2019;303:1–6. PMID: 30794799. doi:10.1016/j.cbi.2019.02.015. 30794799 PMC6743730

[37] Biagi E, Franceschi C, Rampelli S, Severgnini M, Ostan R, Turroni S, Consolandi C, Quercia S, Scurti M, Monti D et al. Gut microbiota and extreme longevity. Curr Biol. 2016;26(11):1480–1485. PMID: 27185560. doi:10.1016/j.cub.2016.04.016. 27185560

[38] Barcena C, Valdes-Mas R, Mayoral P, Garabaya C, Durand S, Rodriguez F, Fernandez-Garcia MT, Salazar N, Nogacka AM, Garatachea N, et al. Healthspan and lifespan extension by fecal microbiota transplantation into progeroid mice. Nat Med. 2019;25(8):1234–1242. PMID: 31332389. doi:10.1038/s41591-019-0504-5. 31332389

[39] Shruthi H, Tamil A, Denise P, Anna L, Susan G, Neil R, Subashan P, Giri N, Aditi G. A molecular index for biological age identified from the metabolome and senescence-associated secretome in humans. Research Square. 2021. PMID:. doi:10.21203/rs.3.rs-62559/v1.

[40] Liu R, Hong J, Xu X, Feng Q, Zhang D, Gu Y, Shi J, Zhao S, Liu W, Wang X et al. Gut microbiome and serum metabolome alterations in obesity and after weight-loss intervention. Nat Med. 2017;23:859–868. PMID: 28628112. doi:10.1038/nm.4358. 28628112

[41] Fletcher JA, Deja S, Satapati S, Fu X, Burgess SC, Browning JD. Impaired ketogenesis and increased acetyl-CoA oxidation promote hyperglycemia in human fatty liver. JCI Insight. 2019;5:e127737. PMID: 31012869. doi:10.1172/jci.insight.127737. PMC6629163 31012869

[42] Surowiec I, Noordam R, Bennett K, Beekman M, Slagboom PE, Lundstedt T, van Heemst D. Metabolomic and lipidomic assessment of the metabolic syndrome in Dutch middle-aged individuals reveals novel biological signatures separating health and disease. Metabolomics. 2019;15(2):23. PMID: 30830468. doi:10.1007/s11306-019-1484-7. 30830468 PMC6373335

[43] Sun Y, Gao H-Y, Fan Z-Y, He Y, Yan Y-X. Metabolomics signatures in type 2 diabetes: a systematic review and integrative analysis. J Clin Endocrinol Metab. 2019;105:1000–1008. PMID:. doi:10.1210/clinem/dgz240. 31782507

[44] Cirulli ET, Guo L, Leon Swisher C, Shah N, Huang L, Napier LA, Kirkness EF, Spector TD, Caskey CT, Thorens, B et al. Profound perturbation of the metabolome in obesity is associated with health risk. Cell Metab. 2019;29(2):488–500.e2. PMID:. doi:10.1016/j.cmet.2018.09.022. 30318341 PMC6370944

[45] Graessler J, Qin Y, Zhong H, Zhang J, Licinio J, Wong ML, Xu A, Chavakis T, Bornstein AB, Ehrhart-Bornstein M, et al. Metagenomic sequencing of the human gut microbiome before and after bariatric surgery in obese patients with type 2 diabetes: correlation with inflammatory and metabolic parameters. Pharmacogenomics J. 2013;13:514–522. PMID: 23032991. doi:10.1038/tpj.2012.43. 23032991

[46] Dao MC. Akkermansia muciniphila abundance is lower in severe obesity, but its increased level after bariatric surgery is not associated with metabolic health improvement. American Journal of Physiology-Endocrinology and Metabolism. 2019 PMID:;317(3):E446–E459. doi:10.1152/ajpendo.00140.2019. 31265324

[47] Zhang H, DiBaise JK, Zuccolo A, Kudrna D, Braidotti M, Yu Y, Parameswaran P, Crowell MD, Wing R, Rittman BE, et al. Human gut microbiota in obesity and after gastric bypass. Proc Natl Acad Sci U S A. 2009;106(7):2365–2370. PMID: 19164560. doi:10.1073/pnas.0812600106. 19164560 PMC2629490

[48] Christensen M, Fadnes D, Røst T, Pedersen E, Andersen JR, Våge V, Ulvik A, Midttun Ø, Ueland P, Nygård O et al. Inflammatory markers, the tryptophan-kynurenine pathway, and Vitamin B status after bariatric surgery. PLOS ONE. 2018;13(2):e0192169. PMID:. doi:10.1371/journal.pone.0192169. 29401505 PMC5798786

[49] Tuomainen M, Lindström J, Lehtonen M, Auriola S, Pihlajamäki J, Peltonen M, Tuomilehto J, Uusitupa M, de Mello VD, Hanhineva K. Associations of serum indolepropionic acid, a gut microbiota metabolite, with type 2 diabetes and low-grade inflammation in high-risk individuals. Nutr Diabetes. 2018;8(1):35. PMID:. doi:10.1038/s41387-018-0046-9. 29795366 PMC5968030

[50] de Mello VD, Paananen J, Lindström J, Lankinen MA, Shi L, Kuusisto J, Pihlajamäki J, Auriola S, Lehtonen M, Rolandsson O, et al. Indolepropionic acid and novel lipid metabolites are associated with a lower risk of type 2 diabetes in the finnish diabetes prevention study. Sci Rep. 2017;7(1):46337. PMID:. doi:10.1038/srep46337. 28397877 PMC5387722

[51] Windey K, De Preter V, Verbeke K. Relevance of protein fermentation to gut health. Mol Nutr Food Res. 2012;56(1):184–196. PMID:. doi:10.1002/mnfr.201100542. 22121108

[52] Li JV, Ashrafian H, Bueter M, Kinross J, Sands C, Le Roux CW. Metabolic surgery profoundly influences gut microbial–host metabolic cross-talk. Gut. 2011;60(9):1214. PMID:. doi:10.1136/gut.2010.234708. 21572120 PMC3677150

[53] West KA, Kanu C, Maric T, McDonald JAK, Nicholson JK, Li JV, Johnson MR, Holmes E, Savvidou MD. Longitudinal metabolic and gut bacterial profiling of pregnant women with previous bariatric surgery. Gut. 2020;69(8):1452. PMID:. doi:10.1136/gutjnl-2019-319620. 31964751 PMC7398482

[54] Nicholson JK, Holmes E, Kinross J, Burcelin R, Gibson G, Jia W, Pettersson S. Host-gut microbiota metabolic interactions. Science. 2012;336(6086):1262–1267. PMID: 22674330. doi:10.1126/science.1223813. 22674330

[55] Suzuki Y, Kido J, Matsumoto S, Shimizu K, Nakamura K. Associations among amino acid, lipid, and glucose metabolic profiles in childhood obesity. BMC Pediatr. 2019;19(1):273. PMID:. doi:10.1186/s12887-019-1647-8. 31387549 PMC6683574

[56] Libert DM, Nowacki AS, Natowicz MR. Metabolomic analysis of obesity, metabolic syndrome, and type 2 diabetes: amino acid and acylcarnitine levels change along a spectrum of metabolic wellness. PeerJ. 2018;6:e5410. PMID: 30186675. doi:10.7717/peerj.5410. 30186675 PMC6120443

[57] Rangel-Huerta OD, Pastor-Villaescusa B, Gil A. Are we close to defining a metabolomic signature of human obesity? A systematic review of metabolomics studies. Metabolomics. 2019;15:93. PMID:. doi:10.1007/s11306-019-1553-y. 31197497 PMC6565659

[58] Lehmann R, Zhao X, Weigert C, Simon P, Fehrenbach E, Fritsche J, Machann J, Schick F, Wang J, Hoene M, et al. Medium chain acylcarnitines dominate the metabolite pattern in humans under moderate intensity exercise and support lipid oxidation. PloS One. 2010;5(7):e11519–e. PMID: 20634953. doi:10.1371/journal.pone.0011519. 20634953 PMC2902514

[59] Ribel-Madsen A, Ribel-Madsen R, Brøns C, Newgard CB, Vaag AA, Hellgren LI. Plasma acylcarnitine profiling indicates increased fatty acid oxidation relative to tricarboxylic acid cycle capacity in young, healthy low birth weight men. Physiological Reports. 2016;4(19):e12977. PMID: 27694528. doi:10.14814/phy2.12977. 27694528 PMC5064135

[60] Rao Y, Kuang Z, Li C, Guo S, Xu Y, Zhao D, Hu Y, Song B, Jiang Z, Ge Z, et al. Gut Akkermansia muciniphila ameliorates metabolic dysfunction-associated fatty liver disease by regulating the metabolism of L-aspartate via gut-liver axis. Gut Microbes. 2021;13(1):1–19. PMID: 34030573. doi:10.1080/19490976.2021.1927633. PMC8158032 34030573

[61] Huo Y, Lu X, Wang X, Wang X, Chen L, Guo H, Zhang M, Li Y. Bifidobacterium animalis subsp. lactis A6 alleviates obesity associated with promoting mitochondrial biogenesis and function of adipose tissue in mice. Molecules. 2020;25. PMID: 32218367. doi:10.3390/molecules25071490. PMC7180933 32218367

[62] Liu J, Li Y, Yang P, Wan J, Chang Q, Wang TTY, Lu W, Zhang Y, Wang Q, Yu LL. Gypenosides reduced the risk of overweight and insulin resistance in C57BL/6J mice through modulating adipose thermogenesis and gut microbiota. J Agric Food Chem. 2017;65:9237–9246. PMID: 28975783. doi:10.1021/acs.jafc.7b03382. 28975783

[63] Masumoto S, Terao A, Yamamoto Y, Mukai T, Miura T, Shoji T. Non-absorbable apple procyanidins prevent obesity associated with gut microbial and metabolomic changes. Sci Rep. 2016;6(1):31208. PMID: 27506289. doi:10.1038/srep31208. 27506289 PMC4979010

[64] Sheng Y, Liu J, Zheng S, Liang F, Luo Y, Huang K, Xu W, He X. Mulberry leaves ameliorate obesity through enhancing brown adipose tissue activity and modulating gut microbiota. Food Funct. 2019;10(8):4771–4781. PMID: 31312821. doi:10.1039/c9fo00883g. 31312821

[65] Gao X, Xie Q, Kong P, Liu L, Sun S, Xiong B, Huang B, Yan L, Sheng J, Xiang H. Polyphenol- and caffeine-rich postfermented pu-erh tea improves diet-induced metabolic syndrome by remodeling intestinal homeostasis in mice. Infect Immun. 2018;86(1). PMID:. doi:10.1128/IAI. PMC5736808 29061705

[66] Anhe FF, Varin TV, Le Barz M, Pilon G, Dudonne S, Trottier J, St-Pierre P, Harris CS, Lucas M, Lemire M, et al. Arctic berry extracts target the gut-liver axis to alleviate metabolic endotoxaemia, insulin resistance and hepatic steatosis in diet-induced obese mice. Diabetologia. 2018;61(4):919–931. PMID: 29270816. doi:10.1007/s00125-017-4520-z. 29270816

[67] Ashrafian F, Shahriary A, Behrouzi A, Moradi HR, Keshavarz Azizi Raftar S, Lari A, Hadifar S, Yaghoubfar R, Ahmadi Badi S, Khatami S, et al. Akkermansia muciniphila-derived extracellular vesicles as a mucosal delivery vector for amelioration of obesity in mice. Front Microbiol. 2019;10:2155. PMID: 31632356. doi:10.3389/fmicb.2019.02155. 31632356 PMC6779730

[68] Song S, Attia RR, Connaughton S, Niesen MI, Ness GC, Elam MB, Hori RT, Cook GA, Park EA. Peroxisome proliferator activated receptor alpha (PPARalpha) and PPAR gamma coactivator (PGC-1alpha) induce carnitine palmitoyltransferase IA (CPT-1A) via independent gene elements. Mol Cell Endocrinol. 2010;325(1–2):54–63. PMID: 20638986. doi:10.1016/j.mce.2010.05.019. 20638986 PMC3160239

[69] Jones PM, Bennett MJ. Chapter 4 - Disorders of mitochondrial fatty acid β-oxidation. In: Garg U, Smith LD, editors. Biomarkers in inborn errors of metabolism. San Diego, USA: Elsevier; 2017. p. 87–101.

[70] Turner N, Cooney GJ, Kraegen EW, Bruce CR. Fatty acid metabolism, energy expenditure and insulin resistance in muscle. Journal of Endocrinology. 2014;220(2):T61–T79. PMID:. doi:10.1530/JOE-13-0397. 24323910

[71] Depommier C, Van Hul M, Everard A, Delzenne NM, De Vos WM, Cani PD. Pasteurized Akkermansia muciniphila increases whole-body energy expenditure and fecal energy excretion in diet-induced obese mice. Gut Microbes. 2020:1–15. PMID: 32167023. doi:10.1080/19490976.2020.1737307. PMC7524283 32167023

[72] Yang Q, Vijayakumar A, Kahn BB. Metabolites as regulators of insulin sensitivity and metabolism. Nat Rev Mol Cell Biol. 2018;19:654–672. PMID:. doi:10.1038/s41580-018-0044-8. 30104701 PMC6380503

[73] Ang QY, Alexander M, Newman JC, Tian Y, Cai J, Upadhyay V, Turnbaugh JA, Verdin E, Hall KD, Leibel RL, et al. Ketogenic diets alter the gut microbiome resulting in decreased intestinal Th17 cells. Cell. 2020;181(6):1263–75.e16. PMID:. doi:10.1016/j.cell.2020.04.027. 32437658 PMC7293577

[74] Muscogiuri G, El Ghoch M, Colao A, Hassapidou M, Yumuk V, Busetto L. European guidelines for obesity management in adults with a very low-calorie ketogenic diet: a systematic review and meta-analysis. Obes Facts. 2021;14(2):222–245. PMID: 33882506. doi:10.1159/000515381. 33882506 PMC8138199

[75] D’Andrea Meira I, Romão TT, Pires do Prado HJ, Krüger LT, Pires MEP, da Conceição PO. Ketogenic diet and epilepsy: What we know so far. Front Neurosci. 2019;13(19):5. PMID: 30760973. doi:10.3389/fnins.2019.00005. 30760973 PMC6361831

[76] Xie G, Zhou Q, Qiu CZ, Dai WK, Wang HP, Li YH. Ketogenic diet poses a significant effect on imbalanced gut microbiota in infants with refractory epilepsy. World J Gastroenterol. 2017;23(33):6164–6171. PMID: 28970732. doi:10.3748/wjg.v23.i33.6164. 28970732 PMC5597508

[77] Olson CA, Vuong HE, Yano JM, Liang QY, Nusbaum DJ, Hsiao EY. The gut microbiota mediates the anti-seizure effects of the ketogenic diet. Cell. 2018;173(7):1728–41.e13. PMID: 29804833. doi:10.1016/j.cell.2018.04.027. 29804833 PMC6003870

[78] Grander C, Adolph TE, Wieser V, Lowe P, Wrzosek L, Gyongyosi B, Ward DV, Grabherr F, Gerner RR, Pfister A, et al. Recovery of ethanol-induced Akkermansia muciniphila depletion ameliorates alcoholic liver disease. Gut. 2018;67:891–901. PMID: 28550049. doi:10.1136/gutjnl-2016-313432. 28550049

[79] Everard A, Plovier H, Rastelli M, Van Hul M, De Wouters D’oplinter A, Geurts L, Druart C, Robine S, Delzenne NM, Muccioli GG, et al. Intestinal epithelial N-acylphosphatidylethanolamine phospholipase D links dietary fat to metabolic adaptations in obesity and steatosis. Nat Commun. 2019;10(1):457. PMID: 30692526. doi:10.1038/s41467-018-08051-7. 30692526 PMC6349942

[80] Kim S, Lee Y, Kim Y, Seo Y, Lee H, Ha J. Akkermansia muciniphila prevents fatty liver, decreases serum triglycerides, and maintains gut homeostasis. Appl Environ Microbiol. 2020;86(7):e03004–19. PMID: 31953338. doi:10.1128/AEM.03004-19. 31953338 PMC7082569

[81] Paoli A, Mancin L, Bianco A, Thomas E, Mota JF, Piccini F. Ketogenic diet and microbiota: friends or enemies? Genes. 2019;10:534. PMID: 31311141. doi:10.3390/genes10070534. PMC6678592 31311141

[82] Ou Z, Deng L, Lu Z, Wu F, Liu W, Huang D, Peng Y. Protective effects of Akkermansia muciniphila on cognitive deficits and amyloid pathology in a mouse model of Alzheimer’s disease. Nutr Diabetes. 2020;10. PMID:. doi:10.1038/s41387-020-0115-8. PMC7176648 32321934

[83] Grajeda-Iglesias C, Durand S, Daillère R, Iribarren K, Lemaitre F, Derosa L, Aprahamian F, Bossut N, Nirmalathasan N, Madeo F, et al. Oral administration of Akkermansia muciniphila elevates systemic antiaging and anticancer metabolites. Aging. 2021;13(5):6375–6405. PMID: 33653967. doi:10.18632/aging.202739. 33653967 PMC7993698

[84] Derrien M, Van Baarlen P, Hooiveld G, Norin E, Muller M, de Vos WM. Modulation of mucosal immune response, tolerance, and proliferation in mice colonized by the mucin-degrader akkermansia muciniphila. Front Microbiol. 2011;2:166. PMID: 21904534. doi:10.3389/fmicb.2011.00166. 21904534 PMC3153965

[85] Zhao S, Liu W, Wang J, Shi J, Sun Y, Wang W, Ning G, Liu R, Hong J. Akkermansia muciniphila improves metabolic profiles by reducing inflammation in chow diet-fed mice. J Mol Endocrinol. 2017;58(1):1–14. PMID: 27821438. doi:10.1530/JME-16-0054. 27821438

[86] Giangregorio N, Palmieri F, Indiveri C. Glutathione controls the redox state of the mitochondrial carnitine/acylcarnitine carrier Cys residues by glutathionylation. Biochimica Et Biophysica Acta (BBA) - General Subjects. 2013;1830(11):5299–5304. PMID:. doi:10.1016/j.bbagen.2013.08.003. 23948593

[87] Babu H, Sperk M, Ambikan AT, Rachel G, Viswanathan VK, Tripathy SP, Nowak P, Hanna LE, Neogi U. Plasma metabolic signature and abnormalities in HIV-infected individuals on long-term successful antiretroviral therapy. Metabolites. 2019;9(10):210. PMID:. doi:10.3390/metabo9100210. PMC6835959 31574898

[88] Manor O, Zubair N, Conomos MP, Xu X, Rohwer JE, Krafft CE, Lovejoy JC, Magis AT. A multi-omic association study of trimethylamine N-oxide. Cell Rep. 2018;24(4):935–946. PMID:. doi:10.1016/j.celrep.2018.06.096. 30044989

[89] Depommier C, Flamand N, Pelicaen R, Maiter D, Thissen JP, Loumaye A, Hermans MP, Everard A, Delzenne NM, Di Marzo V, et al. Linking the endocannabinoidome with specific metabolic parameters in an overweight and insulin-resistant population: from multivariate exploratory analysis to univariate analysis and construction of predictive models. Cells. 2021;10(1):1–19. PMID:. doi:10.3390/cells10010071. PMC7824762 33466285

