# Supplementary material for: Serum metabolite profiling yields insights into health promoting effect of A. muciniphila in human volunteers with a metabolic syndrome
Source: Gut Microbes. 2021 Nov 23;13(1):1994270. doi: 10.1080/19490976.2021.1994270 (PMC8632301; doi:10.1080/19490976.2021.1994270)
Supplement: Supplemental Material [file KGMI_A_1994270_SM3967.zip › supplemental figure 1.pdf]

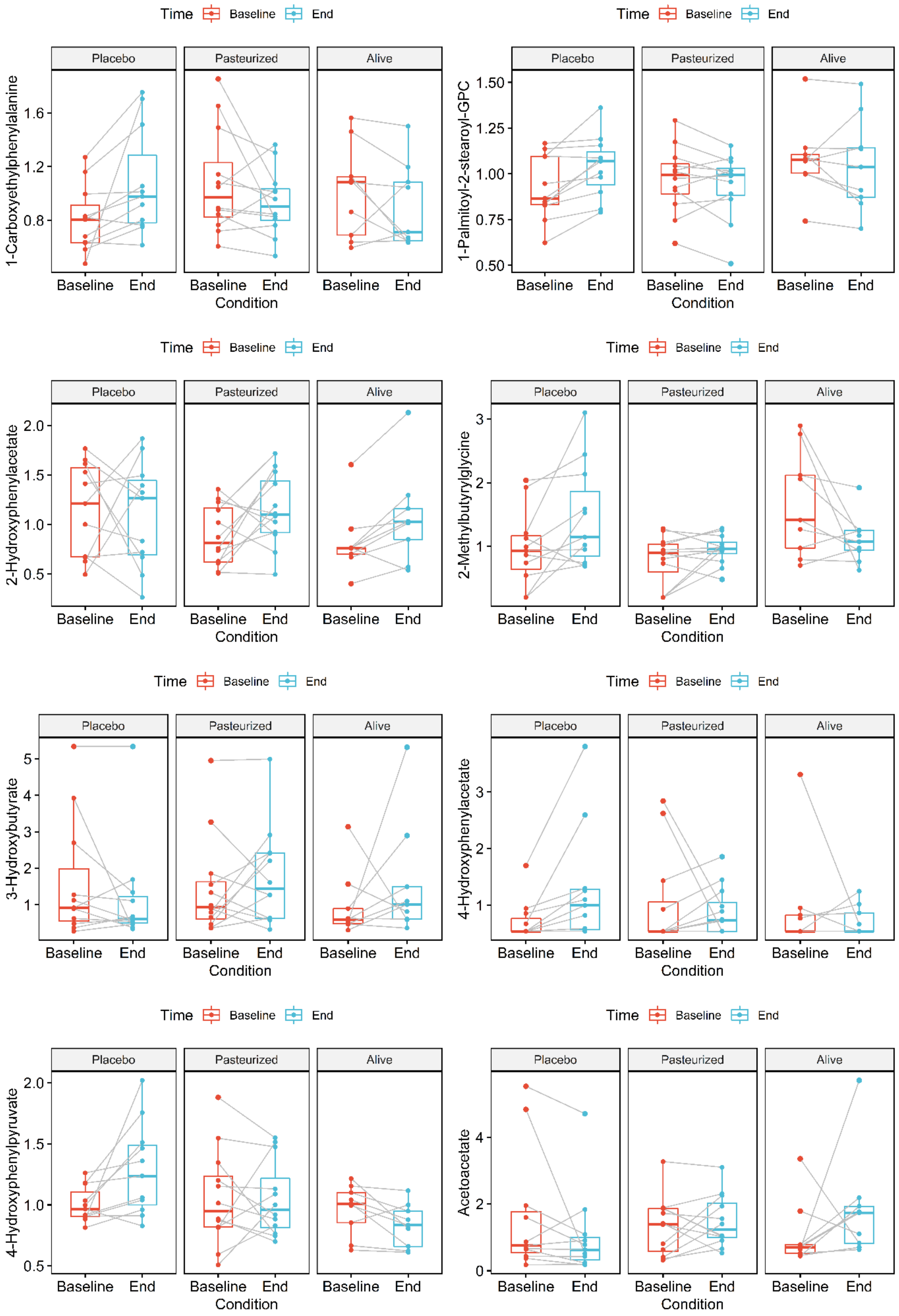
Time Baseline End
Placebo
Pasteurized
Alive
1-Carboxyethylphenylalanine
1-Palmitoyl-2-stearoyl-GPC
2-Hydroxyphenylacetate
2-Methylbutyrylglycine
3-Hydroxybutyrate
4-Hydroxyphenylacetate
4-Hydroxyphenylpyruvate
Acetoacetate
Baseline End
Condition

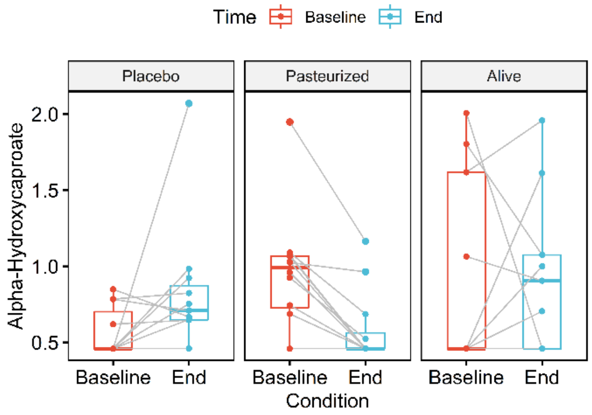
Time Baseline End
Placebo
Pasteurized
Alive
Alpha-Hydroxycaproate
2.0
1.5
1.0
0.5
Baseline End
Condition

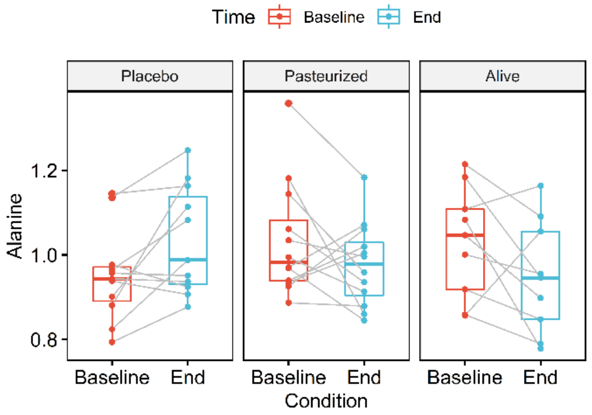
Time Baseline End
Placebo
Pasteurized
Alive
Alanine
1.2
1.0
0.8
Baseline End
Condition

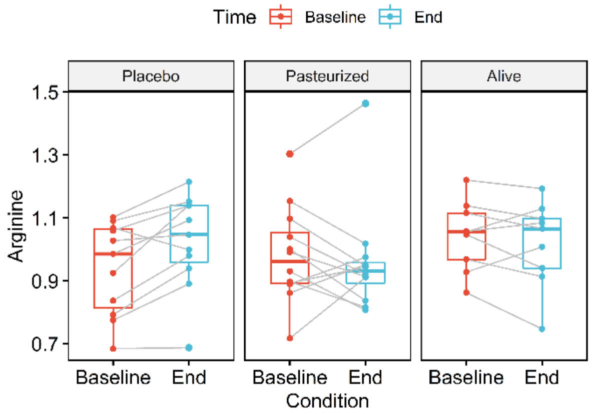
Time Baseline End
Placebo
Pasteurized
Alive
Arginine
1.5
1.3
1.1
0.9
0.7
Baseline End
Condition

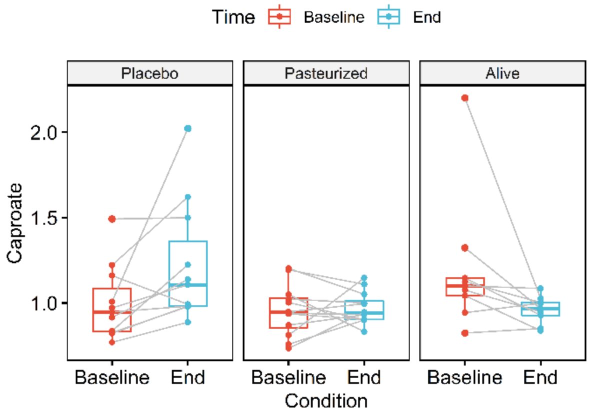
Time Baseline End
Placebo
Pasteurized
Alive
Caproate
2.0
1.5
1.0
Baseline End
Condition

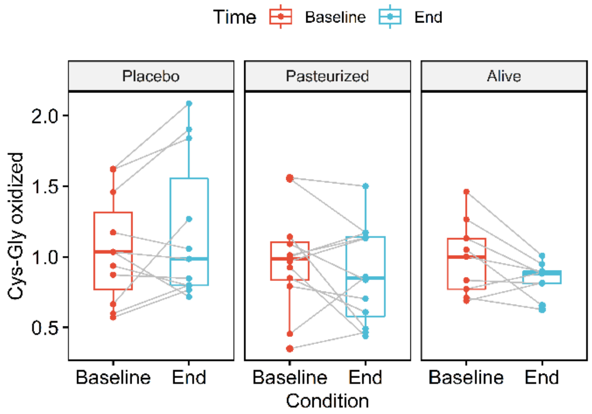
Time Baseline End
Placebo
Pasteurized
Alive
Cys-Gly oxidized
2.0
1.5
1.0
0.5
Baseline End
Condition

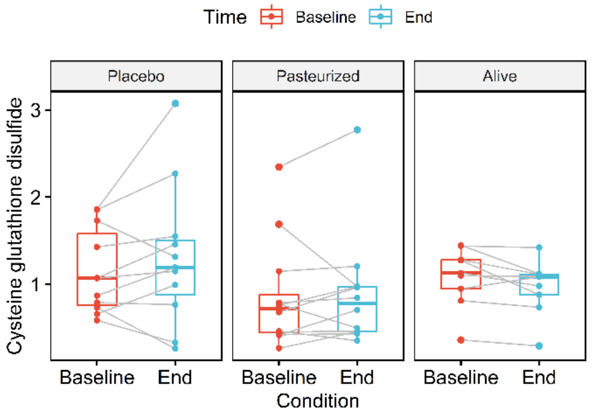
Time Baseline End
Placebo
Pasteurized
Alive
Cysteine glutathione disulfide
3
2
1
Baseline End
Condition

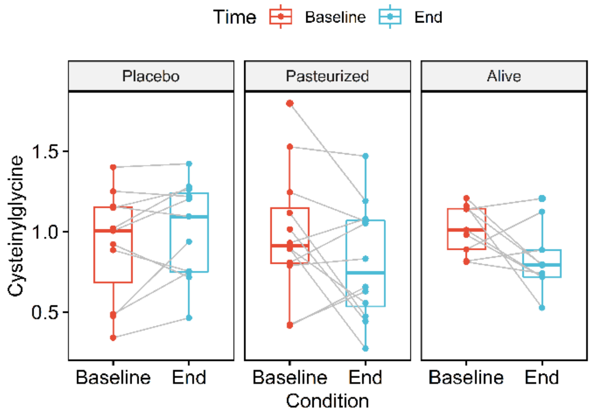
Time Baseline End
Placebo
Pasteurized
Alive
Cysteinylglycine
1.5
1.0
0.5
Baseline End
Condition

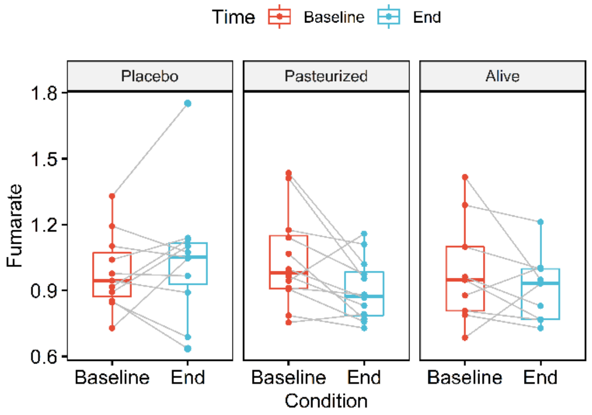
Time Baseline End
Placebo
Pasteurized
Alive
Fumarate
1.8
1.5
1.2
0.9
0.6
Baseline End
Condition

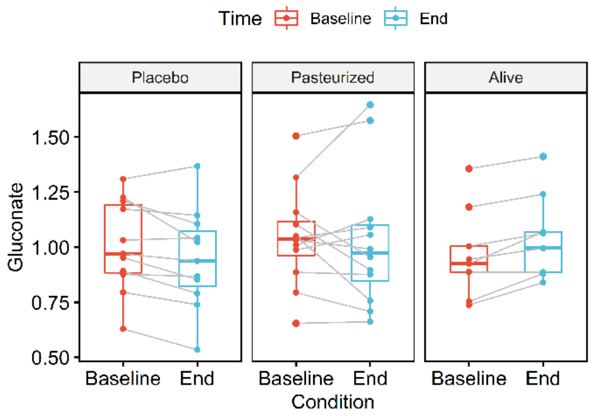
Time Baseline End
Placebo
Pasteurized
Alive
Gluconate
1.50
1.25
1.00
0.75
0.50
Baseline End
Condition

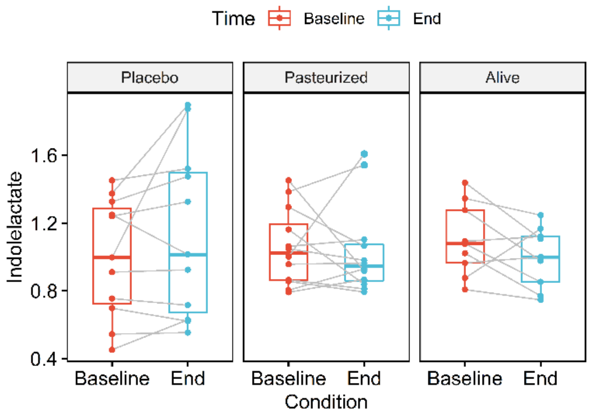
Time Baseline End
Placebo
Pasteurized
Alive
Indolelactate
1.6
1.2
0.8
0.4
Baseline End
Condition

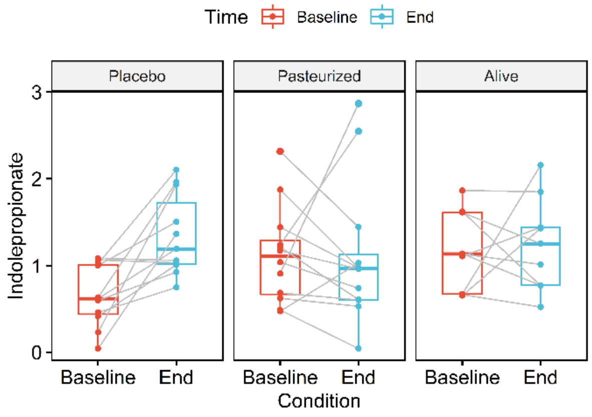
Time Baseline End
Placebo
Pasteurized
Alive
Indolepropionate
3
2
1
0
Baseline End
Condition

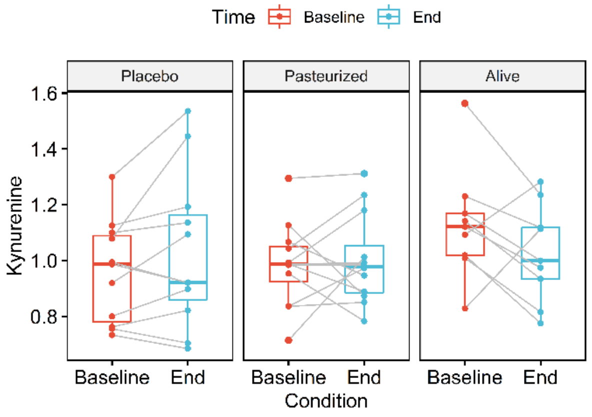
Time Baseline End
Placebo
Pasteurized
Alive
Kynurenine
1.6
1.4
1.2
1.0
0.8
Baseline End
Condition

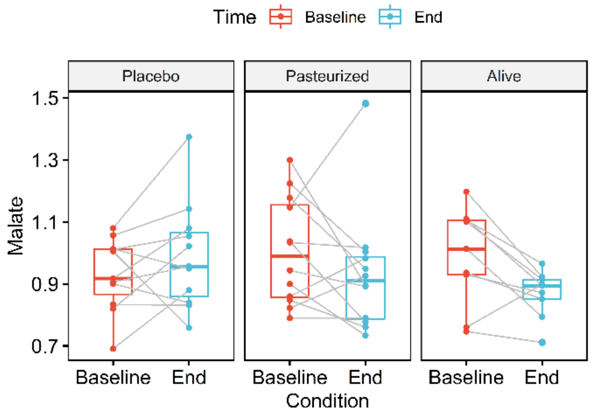
Time Baseline End
Placebo
Pasteurized
Alive
Malate
1.5
1.3
1.1
0.9
0.7
Baseline End
Condition

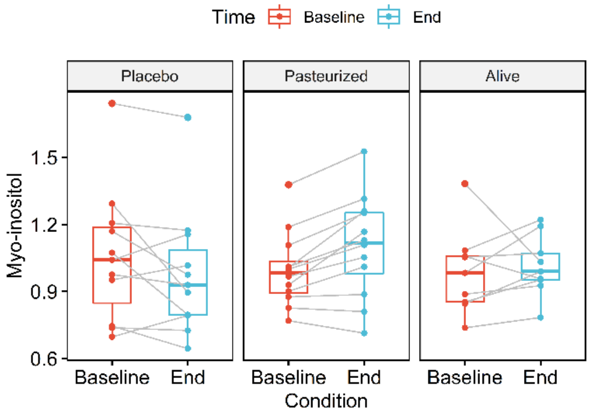
Time Baseline End
Placebo
Pasteurized
Alive
Myo-inositol
1.5
1.2
0.9
0.6
Baseline End
Condition

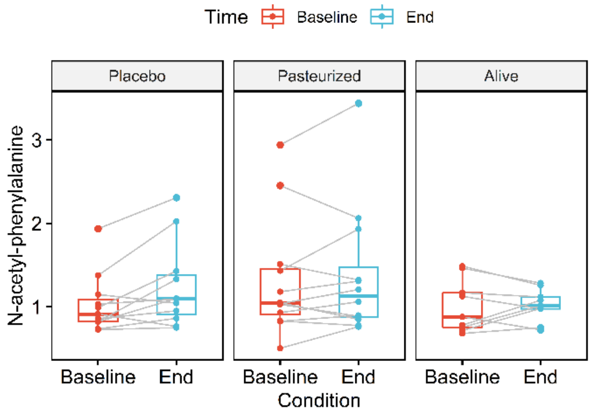
Time Baseline End
Placebo
Pasteurized
Alive
N-acetyl-phenylalanine
3
2
1
Baseline End
Condition

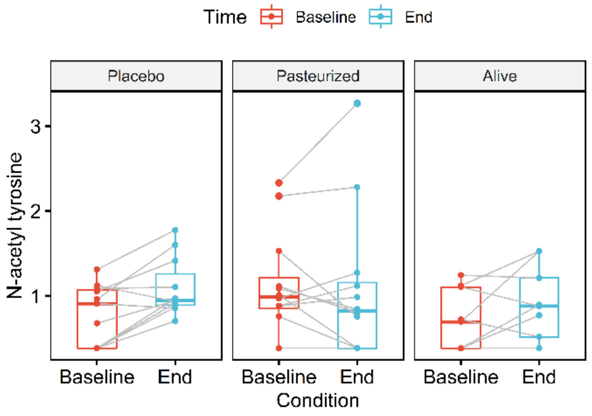
Time Baseline End
Placebo
Pasteurized
Alive
N-acetyl tyrosine
3
2
1
Baseline End
Condition

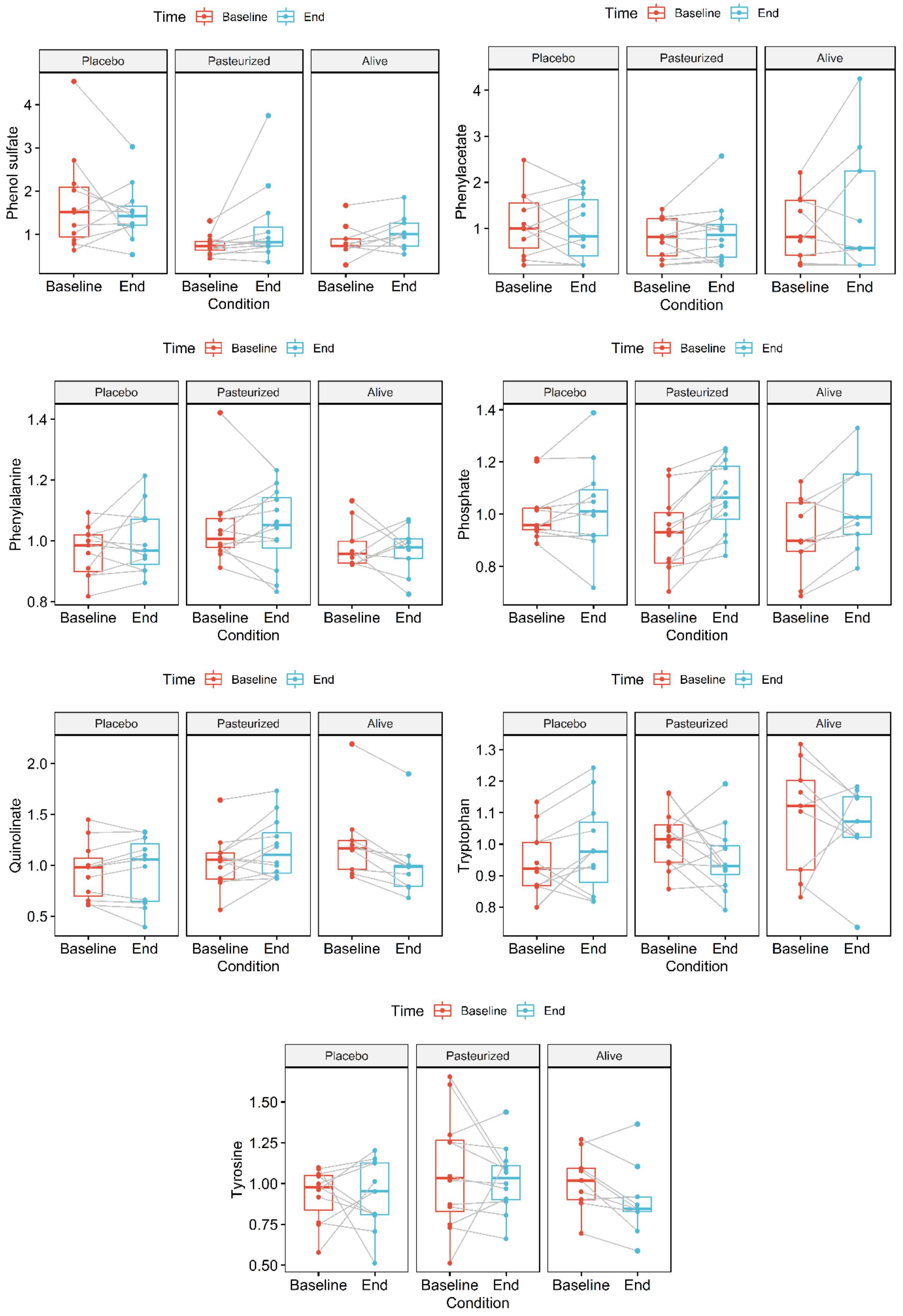

Supplemental Figure 1: Box plots of relative quantification for metabolites of interest in serum, according to the group and time of analysis. Box denotes 25th and 75th percentiles; line within box denotes the median. whisker denotes standard deviation.
