# Supplementary material for: Serum metabolite profiling yields insights into health promoting effect of A. muciniphila in human volunteers with a metabolic syndrome
Source: Gut Microbes. 2021 Nov 23;13(1):1994270. doi: 10.1080/19490976.2021.1994270 (PMC8632301; doi:10.1080/19490976.2021.1994270)
Supplement: Supplemental Material [file KGMI_A_1994270_SM3967.zip › supplementary table.docx]

**Table S1. Serum metabolite changes in response pasteurized *A. muciniphila* supplementation in individuals with metabolite syndrome.**

Top metabolites (P>0.05) revealed by the volcano plot for which the treatment effect was significantly different from the placebo interventional effect. The variables are ordered top-to-bottom as the most-to-least important mean difference from placebo (=Global Delta). The global delta was calculated by subtracting the “mean difference” of the pasteurized group from that of the placebo for each metabolite. Non-parametric two-tailed Mann–Whitney U-tests were used to assess significant differences between the mean differences of the treated groups versus the mean differences of the placebo group, the corresponding p values are indicated in the third column. Numeric values of treatment effect indicated the fold change for each group after median-scaling. Matched pairs t-tests were performed on log-transformed, median-scaled data to evaluate intra-group change. The case color indicates the significance of p values for fold changes greater than 1.00 (red) and less than 1.00 (green). Metabolites that were similarly modulated by both forms are written in italic. Abbreviations: N, Placebo; P, pasteurized.

**Table S2. Serum metabolite changes in response Alive *A. muciniphila* supplementation in individuals with metabolite syndrome.**

Top metabolites (P>0.05) revealed by the volcano plot for which the treatment effect was significantly different from the placebo interventional effect. The variables are ordered top-to-bottom as the most-to-least important mean difference from placebo (=Global Delta). The global delta was calculated by subtracting the “mean difference” of the pasteurized group from that of the placebo for each metabolite. Non-parametric two-tailed Mann–Whitney U-tests were used to assess significant differences between the mean differences of the treated groups versus the mean differences of the placebo group, the corresponding p values are indicated in the third column. Numeric values of treatment effect indicated the fold change for each group after median-scaling. Matched pairs t-tests were performed on log-transformed, median-scaled data to evaluate intra-group change. The case color indicates the significance of p values for fold changes greater than 1.00 (red) and less than 1.00 (green). Metabolites that were similarly modulated by both forms are written in italic. Abbreviations: N, Placebo; A, alive.
